# *PIK3CA* is recurrently mutated in canine mammary tumors, similarly to in human mammary neoplasia

**DOI:** 10.1038/s41598-023-27664-7

**Published:** 2023-01-12

**Authors:** Maja Louise Arendt, Sharadha Sakthikumar, Malin Melin, Ingegerd Elvers, Patricio Rivera, Majbritt Larsen, Sara Saellström, Frode Lingaas, Henrik Rönnberg, Kerstin Lindblad-Toh

**Affiliations:** 1 Department of Veterinary Clinical Sciences, University of Copenhagen, Copenhagen, Denmark; 2 Science for Life Laboratory, Department of Medical Biochemistry and Microbiology, Uppsala University, Uppsala, Sweden; 3 Broad Institute of MIT and Harvard, Cambridge, MA USA; 4 Science for Life Laboratory, Department of Immunology, Genetics and Pathology, Clinical Genomics Uppsala, Uppsala University, Uppsala, Sweden; 5 Karolinska Hospital, Stockholm, Sweden; 6 Vettris Sundsvall, Sundsvall, Sweden; 7 Evidensia Specialist Hospital, Helsingborg, Sweden; 8 Swedish University of Agricultural Sciences, Uppsala, Sweden; 9 Veterinary Faculty, Norwegian University of Life Sciences, Ås, Norway

**Keywords:** Cancer genetics, Cancer models

## Abstract

Biological features of neoplastic disease affecting mammary gland tissue are shared between canines and humans. Research performed in either species has translational value and early phase clinical trials performed in canines with spontaneous disease could be informative for human trials. The purpose of this study was to investigate the somatic genetic aberrations occurring in canine mammary neoplasia by exome capture and next generation sequencing. Based on 55 tumor-normal pairs we identified the *PIK3CA* gene as the most commonly mutated gene in canine mammary tumors, with 25% of samples carrying mutations in this gene. A recurrent missense mutation was identified, p.H1047R, which is homologous to the human *PIK3CA* hotspot mutation found in different types of breast neoplasia. Mutations homologous to other known human mutation hotspots such as the PIK3CA p.E545K and the KRAS p.G12V/D were also identified. We identified copy number aberrations affecting important tumor suppressor and oncogenic pathways including deletions affecting the *PTEN* tumor suppressor gene. We suggest that activation of the *KRAS* or *PIK3CA* oncogenes or loss of the *PTEN* suppressor gene may be important for mammary tumor development in dogs. This data endorses the conservation of cancer across species and the validity of studying cancer in non-human species.

## Introduction

Humans share not only their lifestyle, environment and dietary habits with their dogs, they also share an array of disorders including different types of neoplastic, endocrine, inflammatory and degenerative disease^1–3^. Several studies have shown similarities in the biological behaviour and genetic alterations in neoplastic disease between dogs and humans^4^. This validates the use of the dog as a comparative model for understanding human neoplastic disease and testing new types of therapy with mutual benefit for both species^5^.

The classification of canine mammary tumors encompasses both hyperplastic, benign and malignant tumors^6^. Dogs commonly present with multiple tumors that can have different histopathological diagnosis^7,8^. A study proposed that benign mammary tumors could progress to become malignant as malignant tumors are larger and seen in older individuals. They also found histological evidence of malignant lesions initiating within areas of pre-existing benign lesions^7^. As canine mammary tumors are heterogenous, it is difficult to validate this theory prospectively.

There are many similarities with regards to mammary tumors in dogs and breast neoplasia in humans including risk factors, biological behaviour, hormonal dependencies and patterns of metastasis^7,9^. Despite that the human classification system for breast cancer is not routinely applied to canine malignant mammary tumors, equivalent subtypes have been identified using immunohistochemistry^9^. The rare inflammatory breast carcinoma subtype found in humans is also seen in dogs with similar clinical characteristics and biological behaviour^10^.

Neutering before the third oestrus cycle can reduce the risk of mammary tumors in dogs. Hence in countries where neutering of dogs which are not intended for breeding is common practice, the risk of mammary tumors is reduced compared to countries where fewer animals are neutered^11^. In Sweden routine neutering of female dogs is not common practice, many dogs are registered in the national kennel club and a large proportion of dogs are also insured. These resources have facilitated research into incidence of mammary tumors in different dog breeds and identification of germline predisposing genetic risk factors^3,11,12^. In this context, studies have been performed showing the incidence of mammary tumors in female dogs in Sweden with an overall incidence of 111 cases per 10,000 dog years at risk (DYAR), and a variance between different breeds ranging from 5 to 319 per 10,000 DYAR^11^. A previous GWAS study comparing cases and controls in a high-risk breed identified a germline association to a locus overlying the *CDK5RAP2* gene^3^.

Germline coding mutations in the tumor suppressor genes *BRCA*1 and *BRCA2* have been shown to cause an increased risk of developing breast cancer in humans^13^. In dogs, the presence of variants in these genes has been investigated and though several variants have been identified within both genes, the exact significance in terms of increased risk of developing cancer or functional disruption of the genes has not been investigated in detail^14–20^.

It is important to identify genetic alterations in neoplastic tissue to understand which genetic aberrations are necessary for malignant development, and predict how cells will respond to treatment. In this study we performed exome capture and subsequent sequencing of tumor and normal DNA from a collection of both benign and malignant canine mammary tumors with the view to identify somatic aberrations important for neoplasia and oncogenesis in dogs and to compare these to the mutational spectra in human breast cancer. In addition, we specifically investigated the presence of germline genetic aberrations in the canine *BRCA1* and *BRCA2* gene loci.

## Results

### Mutational hotspots in *PIK3CA* and *KRAS*

In total DNA from 62 tumor-normal pairs were exome captured and sequenced. Normal DNA representing germline was extracted from nucleated white blood cells. Seven tumor-normal pairs were excluded due to too low sequence coverage of the tumor (mean bait coverage < 40×). The remaining 55 T/N pairs originated from 51 individuals as four dogs had more than one tumor sequenced. Of the 55 tumor samples 21 were diagnosed as adenomas, 11 as carcinomas, 12 as mixed tumors and three as hyperplasia. The remaining eight tumor samples did not have a histopathological evaluation (see in Supplementary Table S1). Grading of the malignant tumors according to a standardized system, was not available. The tumors originated from different dog breeds with an overrepresentation of English springer spaniels (ESS, n = 31) and German shepherds (GS, n = 9). The mean bait coverage for the final set of tumor and normal samples was 72.7X (SD 17.9X) and 25.5X (SD 11.6X), respectively.

The number of somatic mutations detected in each tumor ranged from 12 to 268 with a mean of 80.8 (SD 47.5) mutations. This is equivalent to 0.5 mutations per captured Mb (see Supplementary Table S1 for coverage and call summary and Supplementary Table S2 for a list of all somatic variants). After annotation of tumor variants, the non-synonymous coding variants were extracted from the dataset. The mean number of non-synonymous variants was 12.9 per sample (SD 9.7). When evaluating mutational frequency in the full dataset the *PIK3CA c*ame out as significantly mutated after correcting for multiple testing (qglobal_cv) with a *p*-value = 8.8 × 10^−11^.

**Figure 1:**
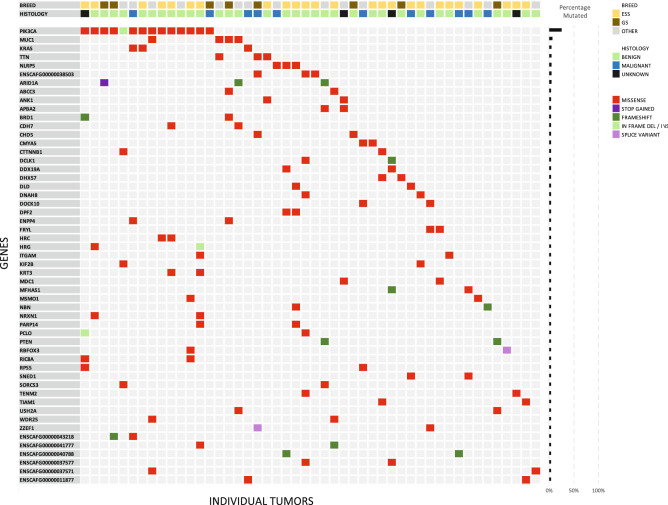
Waterfall plot showing the genes which were mutated in two or more tumors. Fifty-three genes were found to be mutated in more than one tumor sample. Only the fifty tumor samples with overlapping mutations are shown. Five tumors did not show any overlapping mutations and where excluded from the plot. The bar chart to the right illustrates the percentage of tumors carrying a mutation in a particular gene. The percentage is calculated relative to all 55 tumors in the study.

The summary of the recurrently mutated genes is illustrated in the brick plot in Fig. 1. The *PIK3CA* gene was the most frequently mutated gene with 14 tumors (25%) carrying a mutation in this gene. Of these mutations a vast majority (10 of 14) caused the amino acid alteration p.H1047R, homologous to the known *PIK3CA* hotspot mutation found in human breast cancer. One of 14 *PIK3CA* mutations changed the amino acid p.E545D, which occurs at the same location as another known human *PIK3CA* hotspot (p.E545K) mutation. Of the tumors carrying mutations in *PIK3CA,* twelve were classified as benign and 2 were classified as malignant by histology. The *PIK3CA* gene is a well-known oncogene that harbours activating mutations in multiple types of human neoplasia including both benign and malignant breast neoplasia as well as many other epithelial and mesenchymal cancer types such as colorectal carcinomas and angiosarcoma^21,22^.

Another important hotspot mutation was identified in the *KRAS* oncogene. We identified two individuals carrying mutations in the *KRAS* gene homologous to the p.G12D alteration and one individual carrying a mutation homologous to the p.G12V alteration, which have both been found as hotspot alterations in different types of human carcinomas including breast^23^.

### Four genes recurrently mutated in malignant tumors

Focusing on confirmed carcinomas (n = 11) we found four genes which were mutated in more than one sample: *PIK3CA, KRAS, ZZEF1 and DOCK10* were each mutated in two of the carcinomas (18% each). Of the *PIK3CA* mutations one resulted in the H1047R protein alteration and the other resulted in the E545D alteration. In our small dataset of confirmed malignant samples, no genes were discerned as being significantly enriched for mutations.

### Genomic deletions encompass known tumor suppressor genes

**Figure 2:**
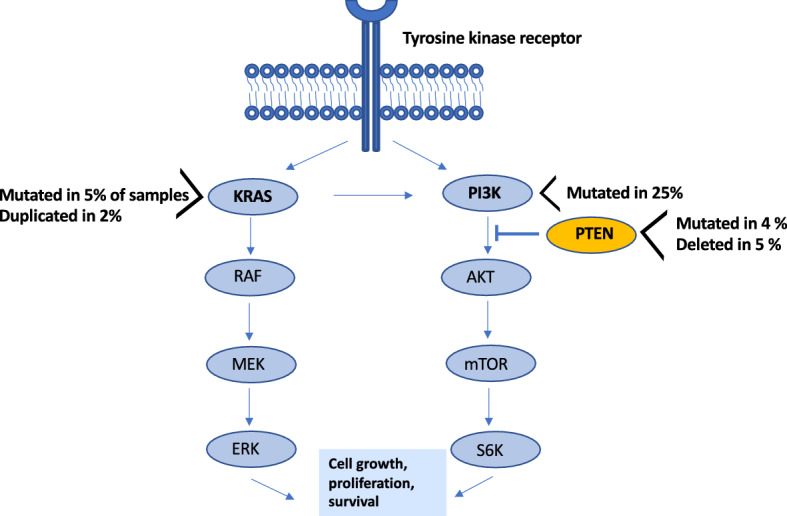
Illustration of the tyrosine receptor pathway activated in canine mammary tumors. Membranous tyrosine kinase receptors such as the HER2 receptor activated the downstream KRAS and PIK3CA pathway leading to downstream cellular proliferations and cell survival. Canine mammary tumors show evidence for activation of PIK3CA and KRAS as well as inactivation of PTEN leading to increased activity of this pathway. Illustration modified from Kim et al. 2020^24^.

Identification of tumor associated copy number alterations can be challenging due to the nature of exome sequencing data. We therefore summarized focal deletions or amplifications overlapping more than 90% of a gene transcript (Supplementary Table S3). We identified deletions in the *PTEN* and the *MYBBP1A* tumor suppressor genes in 5% of tumors. Amplification of the mitogen activated kinase gene *MAPK8* was observed in 5% of samples. We found a single tumor with a duplication of the *KRAS* gene. We did not find any evidence of duplications covering the *ERBB2* (*HER2*) oncogene, which is known to be duplicated in HER2 receptor positive human breast cancer. The HER2 receptor is an activator of the PIK3CA-Akt and RAS dependent pathways and hence though our data does not demonstrate genetic duplication of the *HER2* gene in canine mammary tumors, oncogenic pathways downstream of membranous receptor tyrosine kinases such as HER2 are activated as evidenced by *PIK3CA* and *KRAS* activating mutations and *PTEN* and *KRAS* copy number alterations (Fig. 2)^24,25^.

### Comparison of multiple tumor-normal pairs from the same individuals

It is common for dogs to develop multiple mammary tumors^7,8^. Though mammary tumors can spread locally, one can speculate that multiple mammary tumors are a result of independent events in a predisposing setting, as they can have different histopathological classifications^7,8^. In our dataset we had four individuals which had two tumors each with different histopathological classification. None of the tumor pairs consisted of paired malignant tumors. We evaluated if we could identify any overlapping somatic mutations between the intra-individual tumors. We did not find any overlapping mutations between the paired tumors, which is in agreement with their histopathological differences and that they likely represent independent events. Investigation of a larger dataset based on dogs with multiple malignant tumors is necessary to determine if multiple malignant tumors carry the same cancer driving mutations.

### Multiple germline variants in the *BRCA1* and *BRCA2* genes

From the normal data we identified 10 germline variants located in the coding sequence of the *BRCA1* gene (Supplementary Table S4). Of these variants, one chr9:19,988,291 has previously been reported in a publication by Borge et al.^15^ in dogs. Eight of these variants were missense, one was synonymous and one was classified as a frameshift. Interestingly, four ESS and one cocker spaniel were affected by two missense variants with a SIFT score below 0.05 and one frameshift variant^26^. Both are breeds with an increased risk of developing mammary tumors^11^. No segregation of specific tumor types was found within these carriers.

We identified 18 variants affecting the coding sequence of the *BRCA2* gene. We found 13 missense variants, three synonymous variants, one in-frame deletion and one in-frame insertion. The majority of these germline variants (13/18) have previously been described^15,16,20,27^. Four of the missense variants had a SIFT score equal to or less than 0.05.

PhyloP scores were evaluated for each variant position as a mean to measure evolutionary constraint and functional potential^28^. Three variants in the *BRCA2* gene had a phyloP score > 3. Two of these also had a SIFT score < 0.05 suggesting that these variants could have a functional impact on the *BRCA2* gene. A summary of the identified variants in the *BRCA1* and *BRCA2* genes, allele frequencies, SIFT and phyloP scores can be found in Supplementary Table S4. We lifted over the identified canine *BRCA1* and *BRCA2* variants to the human genome and evaluated each position in the ClinVar database^29^. None of our variants were homologous to known breast cancer risk variants in humans and were all classified as variants of unknown significance^29^.

## Discussion

In this study we identified the *PIK3CA* gene as being the most commonly mutated gene in canine mammary tumors. The majority (10/14) of the mutations resulted in the p.H1047R amino acid change homologous to the human mutational hotspot in this gene. We also identified a single sample having a mutation causing alteration of amino acid 545 (p.E545D), akin to the human hotspot p.E545K. These alterations are known hotspot mutation positions within human neoplasia of both epithelial and mesenchymal origin. Other studies have previously identified the p.H1047R hotspot mutation in canine tumors including glioma, hemangiosarcoma and malignant and benign mammary tumors^30–32^. In our study however, we found that the majority of tumors (12/14) carrying this mutation were benign. This suggests that this mutation could be important for early cellular proliferation and neoplastic initiation but that additional enabling factors or mutations are necessary for malignant transformation of the mammary tissue. The *PIK3CA* gene is commonly mutated in human breast cancer, however it is also seen to be mutated in benign breast lesions such as hyperplasia and adenomas^33,34^. It has also been speculated that *PIK3CA* mutations in human breast tissues are important in early stages of disease whilst the mutation provides less of a selective advantage in later stages^33^. A tumor initiating role for the p.PIK3CA H1047R variant has been demonstrated in mouse breast tumor models and functionally the mutation has been shown to increase the PIK3CA enzyme activity in vivo^35,36^. The prognostic role of *PIK3CA* mutations in breast cancer is equivocal, however a meta-analysis performed on *PIK3CA* mutation status in human breast cancer showed that mutations in this gene represented an independent negative prognostic factor with a hazard ratio of 1.67^37^. From the Catalogue Of Somatic Mutations in Cancer (COSMIC) database it is evident that *PIK3CA* is mutated less frequently in more aggressive breast cancer subtypes. Only 14% of triple negative breast cancers carry this mutation whilst it is present in 32% of estrogen and progesterone receptor positive cancers. It therefore seems to be more common within less aggressive breast cancer, even though it is a negative prognostic indicator within specific breast cancer subtypes^37,38^.

PIK3CA inhibitors, such as alpelisib, are being developed for treating specific subgroups of human breast cancer, such as HER2 negative, *PIK3CA* mutation positive advanced stage breast cancer^39,40^. With the homologous *PIK3CA* mutations found in canine mammary neoplasia, investigation of drug effect and mechanisms of resistance could be performed comparatively in this species, though more studies are needed to investigate the translational use of this type of treatment.

We also identified mutations located in two other known human mutation hotspots, namely mutations equivalent to the PIK3CA p.E545K and the KRAS p.G12V/D. This shows additional similarities between the human and canine disease and the conserved role for these mutational hotspots across species.

### Malignant versus benign neoplasia

Due to the small number (n = 11) confirmed malignant tumors in this study, we could not assess which genes were needed for malignant transformation. Activating hotspot mutations in both the *KRAS* and *PIK3CA* oncogenes were present in both benign and malignant tumors. Though the *PIK3CA* gene is known to be mutated in benign human tumors, we speculate that the *KRAS* mutations could contribute to malignant transformation. *KRAS* mutations affecting codon 12 are reported in human breast cancer and described as putative driver mutations^23^.

### Germline BRCA1 and BRCA2 variants

We identified multiple germline variants within the *BRCA1* and *BRCA2* genes. Germline mutations in these genes in humans have been implicated in increasing the lifetime-risk of developing breast cancer. We did not identify any mutations which were directly homologous to the human *BRCA1* and *BRCA2* germline variants which have been confirmed to increase the risk of cancer in humans, however we did find variants which potentially could influence the function of the BRCA1 and BRCA2 proteins. Of particular interest were several identified variants with high phyloP scores, suggesting that these variants affect highly constrained regions of the gene and could be consequential for protein function. Further, two of the identified variants affecting the BRC repeat 3 domain of the BRCA2 protein (pT1425P and pK1435R) have been shown by functional validation in vitro, to interfere with BRCA2’s interaction with RAD51 which is important for homologous recombination and DNA repair^16^. Both of these variants had low SIFT scores of 0.0 and 0.07 respectively, moderate to high phyloP scores of 3.66 and 1.33, and were present in our dataset with allele frequencies of 0.05 and 0.25. The moderate to high phyloP scores indicate that these mutations are located in positions that are functional hence evolutionarily constrained. Both of these variants are classified as variants of unknown significance according to the ClinVar database suggesting that a well-established role for these mutations in human disease is yet to be established^29^. The p.M332IK BRCA2 insertion has previously been reported, and in vitro functional studies have been performed showing that this variant can affect the nuclear localization signal of the BRCA2 protein^20^. Though we find some functional evidence for a couple of the reported variants, large epidemiological and more elaborate functional studies are needed to evaluate if variants truly increase the risk of mammary neoplasia in canines.

### Study limitations

There are some limitations to the data presented, mainly due to low sequence coverage of the normal samples and insufficient enrichment of the exome regions. We modified the data filtering to allow for detection of true positive variants and added additional conservative filters to minimize false positive calling. We did not have the histopathological diagnosis available for all tumor samples and sequenced tissue was not microdissected from the pathologically classified tissue. Though the tissues for sequencing were sampled adjacent to tissue sent for histopathological classification, we know that canine mammary tumors can be heterogeneous. Hence, the sequenced tissue might in some instances not be representative of the pathological diagnosis, which could explain why very few mutations were identified in some tumors.

## Conclusion

In this study we identified mutations in three known human mutation hotspots PIK3CA p.H1047R, PIK3CA p.E545D and KRAS p.G12V/D, which have all previously been shown to be implicated in human breast neoplasia and even other types of carcinomas in humans. This finding is interesting as it shows that the evolutionary biology of neoplasia is at least partially conserved between species, which is likely due to these mutations causing the most advantageous phenotype for the neoplastic cells. This further emphasizes the value of studying cancer comparatively across species to evaluate driving mutations and possible treatment targets. Though our data represents a relatively small cohort of tumor samples, we have found suggestive evidence that for some targeted drugs, such as PIK3CA inhibitors, there could be value in using dogs with naturally occurring mammary tumors as comparative models. Sequencing of a larger population of canine mammary tumors is needed to truly be able to segregate these tumors into comparable human subtypes and to understand the full comparative potential.

## Materials and methods

### Patient samples

Mammary tumor tissue samples and peripheral EDTA blood samples were collected from canine patients which were undergoing surgery as treatment for their disease at veterinary clinics within Sweden and Norway. The mammary tumor tissues collected, consisted of surplus diagnostic material collected without compromising the diagnosis and histopathological evaluation for the patient. The surgical treatment was performed whilst the patient was anesthetized with an inhalation anaesthetic (sevoflurane or isoflurane). Tissue samples were stored in RNAlater and an adjacent piece of tissue was sent for histopathological evaluation together with the main tumor. RNAlater embedded tissues were stored at room temperature for approximately 24–72 h before storing at − 80 °C. EDTA blood was aliquoted and frozen at − 80 °C within 1–5 days of collection.

### Ethical approval

All samples were collected with the owners’ written informed consent and in agreement with Ethical guidelines. Ethical approval was granted by the regional animal ethics committee (Uppsala ethics committee on animal experiments/ Uppsala djurförsöksetiska nämnd: Dnr C12/15, D318/9, C139/9). All methods involving animal tissues are reported in accordance with the ARRIVE guidelines. All methods are carried out in accordance with relevant guidelines and regulations.

### DNA extraction

Blood DNA was extracted using either the QIASymphony robot (Qiagen) together with the QIASymphony DNA Midi kit (Qiagen) or manually by NucleoSpin Blood, Mini DNA extraction kit (Macherey–Nagel). Tissue DNA was extracted using the AllPrep DNA/RNA/miRNA extraction kit (Qiagen). Tissue pieces stored in RNAlater were homogenized in RLT buffer using the BeadBeater (Biospec) with zirconia beads and after cell lysis the RNA and DNA containing fractions were separated. Concentration and purity were measured using Qubit Fluorometric Quantification (ThermoFisher).

### Exome library preparation and DNA sequencing

Exome library preparation was performed using the 140702_canFam3_exomeplus_BB_EZ_HX1 liquid exome capture (Roche), using a custom capture protocol. Each library was individually barcoded and sequenced using the Illumina NovaSeq™ 6000 sequencing system with 150 bp paired end sequencing reads.

### Data analysis

DNA sequences were aligned by following the GATK 3.8–0 best practices recommendations for tumor-normal data^41^. Fastq files were aligned to CanFam3.1 using BWA MEM 0.7.17^42^. Conversion of sam to bam files was performed using samtools 1.9^43^. Duplicates were marked using picard 2.10.3. Further base recallibration was performed using GATK 3.8–0. Sequence bait capture coverage was calculated using Picard 2.10.3 (CollectHSMetrics). Further quality of the aligned data and calculation of coverage and standard deviation was performed using Qualimap^44^.

Somatic variants were called using GATK (3.8–0) Mutect 2^45^. Due to the relatively low coverage for some of the normal samples, custom settings were applied to reduce the levels of false positive and false negative genetic variants detected. This included lowering the pool of normal (PON) threshold to 1 and setting the Nlod to 0. Further any variants present in two separate normal canine variant databases were removed as being potential germline variants^46,47^. Variants were excluded if there was ≤ 2 alternative reads supporting the alternative allele or if the coverage was more than the mean + 5xSD. This was done to filter out possible alignment errors. Finally, if any reads were found to support the alternative allele in the matched normal sample the variant was filtered out. The final list of somatic variants was annotated, using VEP v.99^26,48^. Recurrently mutated genes were defined as genes which were mutated in at least two tumor samples. Genes which were mutated more than once in the same individual were not interpreted as recurrently mutated.

### Detection of genes under positive selection using dNdScv

Evaluation of genes under positive selection as putative oncogenic drivers was performed using the tool *dNdScv*^49^*.* Genes with a *p*-value of less than 0.05 after adjusting for multiple testing were considered significant.

### Copy number aberrations

Evaluation of copy number aberrations was performed using CNVkit in tumor normal mode using the CNVkit pipeline^50^. Alterations were sorted according to size. Annotation was performed using VEP v 99^48^. Based on the length of the observed genomic alterations, copy number events were either classified as focal- or broad/large-scale changes. Regions where the fold change (either amplification or deletion) ranged from 1 to 250 kb, were demarcated as focal copy number events. Large-scale changes were delineated if the copy number event covered > 250 kb. Focal changes may result from positive selection events during oncogenesis; therefore, the genes perturbed in these regions were ascertained. The copy number altered genomic regions were annotated with gene information using the utility BEDTools^51^. Genes where 90% of their genomic coordinates overlapped with the focal changes were extracted and tabulated as a high-confidence dataset and examined further.

### Identification of *BRCA1 *and *BRCA2* germline variants

Germline variants located within the *BRCA1* and *BRCA2* genes were extracted from the 51 normal samples and annotated using VEP v 99^26,48^. Identified variants were compared to already known canine variants reported in published literature and the phyloP score was evaluated for each variant^28^.

## Supplementary Information

Supplementary Information 1.

Supplementary Information 2.

Supplementary Information 3.

Supplementary Information 4.

## Data Availability

Sequencing and metadata is available through the European Nucleotide Archieve : Project : PRJEB53653, link to project archive https://www.ebi.ac.uk/ena/browser/view/PRJEB53653.
