# Supplementary material for: PIK3CA is recurrently mutated in canine mammary tumors, similarly to in human mammary neoplasia
Source: Sci Rep. 2023 Jan 12;13:632. doi: 10.1038/s41598-023-27664-7 (PMC9837039; doi:10.1038/s41598-023-27664-7)
Supplement: Supplementary file 1 — Supplementary Information 1. [file 41598_2023_27664_MOESM1_ESM.pdf]

ID	BREED	HISTOLOGY	SUBCLASS	Mean bait coverage T	Mean bait coverage N	Number of somatic variants	Number Nonsynonumous SNVs
1	ESS	Missing		61.20	40.3	49	5
2	ESS	Missing		71.60	35.8	16	2
3	ESS	Adenoma	Simple	54.9	49.2	12	6
4	ESS	Adenoma	Simple	49	32	24	2
5	ESS	Adenoma	Complex	52.1	28.2	25	2
6	ESS	Adenoma	Comlpex	76.3	38.8	47	7
7	ESS	Mixed tumor		78.3	36.1	147	22
8	ESS	Adenoma	Complex	62.7	32.8	48	1
9	ESS	Carcinoma	Squamous	54	31.3	57	9
10	ESS	Hyperplasia	Adenosis	75.2	31.3	124	20
11	ESS	Adenoma	Simple	65.5	37.4	51	6
12	ESS	Missing		73.9	33.6	64	19
13	ESS	Mixed tumor		65.9	31.6	58	10
14	ESS	Adenoma	Complex	57.9	31.6	75	8
15	ESS	Adenoma	Simple	73.8	42.8	81	11
16	ESS	Carcinoma	Simple	75.8	29.6	43	5
17	GS	Carcinoma	Simple	61.1	27.5	108	15
18	GS	Adenoma	Complex	77	38.5	92	21
19	GS	Hyperplasia	Lobular	58.2	35.6	91	14
20	GS	Adenoma	Complex	68.8	34.2	64	14
21	LAJKA	Carcinoma		72	38.1	130	19
22	SHETLAND SHEEPDOG	Carcinoma	Simple	76.6	37.1	142	27
23	DACHSHUND	Adenoma	Tubulopapill	68.2	30.3	80	15
24	BEDLINGTON TERRIER	Carcinoma	Tubulopapill	76.9	32.9	72	18
25	FIELD SPANIEL	Adenoma	Complex	76.4	38.6	79	12
26	NOVA SCOTIA RETRIEVER	Adenoma	Complex	74.8	13.7	88	12
27	CKCS	Carcinoma		61.3	13.2	86	11
28	AMERICAN COCKER SPANIEL	Adenoma	Simple	67.2	17.5	68	5
29	CS	Carcinoma	Simple	69.9	18	92	6
30	ENGLISH SETTER	Carcinoma	Complex	81.8	13.6	167	26
31	DACHSHUND	Carcinoma	Mucin rich	52.4	13.8	95	15
32	COTON DE TULEAR	Adenoma		75.1	18.5	141	21
33	CS	Missing	Missing	65.4	17.1	98	16
34	ESS	Mixed tumor		71.1	16.4	57	9
35	ESS	Adenoma	Complex	56.7	15.1	42	4
36	ESS	Mixed tumor		59.5	13.8	37	5
37	ESS	Mixed tumor		81.1	15.4	67	13
38	ESS	Mixed tumor		65.4	20.2	59	9
39	ESS	Adenoma	Complex	43.6	10	32	9
40	ESS	Missing	Missing	64	15.1	54	10
41	ESS	Mixed tumor		69.5	13.3	89	15
42	ESS	Missing		58.1	12.7	44	12
43	GS	Missing		65.9	16.8	70	7
44	CS	Adenoma	Complex	64.8	9.2	86	14
45	GS	Hyperplasia	lobular	87.3	9.3	77	17
46	GS	Adenoma	Complex	88.3	9.3	240	68
47	CS	Mixed tumor		104.3	17.8	131	20
48	ESS	Missing		55.9	10.8	37	6
49	ESS	Mixed tumor		73.4	14.3	66	10
50	ESS	Mixed tumor		112.5	13.2	92	13
51	ESS	Adenoma	Complex	103.3	14.3	95	14
52	ESS	Mixed tumor		137.3	41.8	76	12
53	ESS	Mixed tumor		127.8	45.2	268	21
54	GS	Carcinoma		78	40.2	53	9
55	GS	Adenoma	Complex	101	40.2	59	12

Supplementary Table S1) Summary of samples included in the study. Table shows sample id, histological diagnosis and subclassification, mean tumor and normal sample sequencing coverage, the total number of somatic variants detected after filtration and the number of non-synonymous variants detected. Non-synonymous mutations in this table include nonsense mutations and splice-site variants. Breed ESS=English springer spaniel, GS=German Shepherd, CKCS= Cavalier king Charles spaniel, CS= cocker spaniel. Tumors 9-10, 13-14, 45-46 and 54-55 share the same normal.
