# Supplementary material for: PIK3CA is recurrently mutated in canine mammary tumors, similarly to in human mammary neoplasia
Source: Sci Rep. 2023 Jan 12;13:632. doi: 10.1038/s41598-023-27664-7 (PMC9837039; doi:10.1038/s41598-023-27664-7)
Supplement: Supplementary file 3 — Supplementary Information 3. [file 41598_2023_27664_MOESM3_ESM.pdf]

Supplementary Table S3

Sample	Chromosome	Gene_Start	Gene_Stop	Gene_Name	Length_Amplified	%_of_Gene_Amplified
TN-1	chr8	26637895	26717781	SOS2	79886	100
TN-1	chr9	10277802	10280466	ENSCAFG00000042619	2664	100
TN-1	chr9	10282539	10283222	ENSCAFG00000015156	683	100
TN-1	chr9	10290616	10330701	ENSCAFG00000028642	40085	100
TN-1	chr13	58911361	58932913	ENSCAFG00000029376	21552	100
TN-2	chr2	4474618	4475949	ENSCAFG00000049457	1331	100
TN-2	chr2	4580418	4602381	ENSCAFG00000023536	21963	100
TN-2	chr2	36378640	36381614	PCDHGB5	2974	100
TN-2	chr5	32878338	32888316	ALOX15B	9978	100
TN-2	chr5	42143474	42169517	COPS3	26043	100
TN-2	chr8	2210536	2212731	ENSCAFG00000049482	2100	96
TN-2	chr8	2233607	2234643	ENSCAFG00000047976	1036	100
TN-2	chr8	2273563	2274464	ENSCAFG00000048830	901	100
TN-2	chr8	2282856	2283504	ENSCAFG00000029069	648	100
TN-2	chr8	2289879	2290968	ENSCAFG00000048140	1089	100
TN-2	chr8	2295396	2380876	ENSCAFG00000045242	85480	100
TN-2	chr8	2304639	2305537	ENSCAFG00000014973	898	100
TN-2	chr8	2311539	2312292	ENSCAFG00000024337	753	100
TN-2	chr8	2323009	2323657	ENSCAFG00000028548	648	100
TN-2	chr8	2356960	2358745	ENSCAFG00000031023	1785	100
TN-2	chr8	2365535	2366207	ENSCAFG00000043621	672	100
TN-2	chr8	2370648	2371741	ENSCAFG00000043519	1093	100
TN-2	chr8	2372687	2375185	ENSCAFG00000049498	2498	100
TN-2	chr8	2406019	2408341	ENSCAFG00000046168	2322	100
TN-2	chr8	2411890	2412979	ENSCAFG00000047752	1089	100
TN-2	chr8	2418492	2418979	ENSCAFG00000014977	487	100
TN-2	chr9	10277802	10280466	ENSCAFG00000042619	2664	100
TN-2	chr9	10282539	10283222	ENSCAFG00000015156	683	100
TN-2	chr9	10290616	10330701	ENSCAFG00000028642	40085	100
TN-2	chr9	32461679	32462140	CCDC182	461	100
TN-2	chr10	11262265	11292832	CPSF6	30084	98
TN-2	chr20	22888270	22890284	ENSCAFG00000006577	1933	96
TN-2	chr20	54224789	54225841	FUT5	1052	100
TN-2	chr37	7169192	7216590	BOLL	43306	91

TN-2	chr38	23205769	23231158	ENSCAFG00000043714	25215	99
TN-2	chrX	61980167	61982446	ENSCAFG00000046786	2279	100
TN-2	chrX	105685976	105686988	ENSCAFG00000041731	1012	100
TN-3	chr18	49090689	49104051	MRPL21	13362	100
TN-3	chr37	12086749	12140228	CYP20A1	53479	100
TN-4	chr2	4580418	4602381	ENSCAFG00000023536	21963	100
TN-4	chr2	35346168	35346326	IGIP	158	99
TN-4	chr5	42143474	42169517	COPS3	26043	100
TN-4	chr8	2176546	2177259	ENSCAFG00000044478	713	100
TN-4	chr8	2210536	2212731	ENSCAFG00000049482	2195	100
TN-4	chr8	2233607	2234643	ENSCAFG00000047976	1036	100
TN-4	chr8	2273563	2274464	ENSCAFG00000048830	901	100
TN-4	chr8	2282856	2283504	ENSCAFG00000029069	648	100
TN-4	chr8	2289879	2290968	ENSCAFG00000048140	1089	100
TN-4	chr8	2304639	2305537	ENSCAFG00000014973	898	100
TN-4	chr8	2311539	2312292	ENSCAFG00000024337	753	100
TN-4	chr8	2323009	2323657	ENSCAFG00000028548	648	100
TN-4	chr9	54802214	54810039	SPOUT1	7825	100
TN-4	chr16	10449353	10450970	ENSCAFG00000024751	1617	100
TN-4	chr16	13811208	13822017	PGBD2	10809	100
TN-4	chr16	13829171	13884431	ENSCAFG00000043982	55260	100
TN-4	chr16	13947830	13948339	ENSCAFG00000049312	509	100
TN-4	chr16	13991368	13992180	ENSCAFG00000046117	812	100
TN-4	chr16	14002066	14046567	ENSCAFG00000032319	44501	100
TN-4	chr22	32581825	32664714	RBM26	82889	100
TN-4	chr22	32704437	32787151	NDFIP2	82714	100
TN-4	chr23	36922994	36948975	SLC25A36	24073	93
TN-4	chr25	34710934	34721006	ENSCAFG00000042909	10072	100
TN-4	chr25	35011861	35016605	POLR3D	4474	94
TN-4	chr25	35020751	35087545	BMP1	66794	100
TN-4	chr25	35026692	35037163	PHYHIP	10471	100
TN-4	chr25	35088279	35091457	SFTPC	3178	100
TN-4	chr25	35097566	35105940	LGI3	8374	100
TN-4	chr25	35109236	35117048	REEP4	7812	100
TN-4	chr25	35119784	35135545	HR	15761	100
TN-4	chr25	35140969	35143063	NUDT18	2094	100
TN-4	chr25	35146390	35162394	FAM160B2	16004	100
TN-4	chr25	35165876	35176754	DMTN	10878	100
TN-4	chr25	35194394	35199792	FGF17	5398	100
TN-4	chr25	35204906	35226195	ENSCAFG00000009751	21289	100
TN-4	chr25	35233034	35277124	XPO7	43156	98
TN-4	chr25	37250775	37257199	ZNF596	6424	100

TN-4	chr25	44977224	45008538	ENSCAFG00000011833	31314	100
TN-4	chr25	45015723	45021560	ENSCAFG00000044804	5837	100
TN-4	chr25	45032130	45160941	UGT1A6	128811	100
TN-4	chr25	45143564	45144280	ENSCAFG00000011889	716	100
TN-4	chr25	48378970	48410104	RBM44	31134	100
TN-4	chr25	48437919	48466070	RAMP1	28151	100
TN-4	chr25	48503833	48558986	UBE2F	55153	100
TN-4	chr36	10601280	10601561	ENSCAFG00000041552	281	100
TN-5	chr16	10449353	10450970	ENSCAFG00000024751	1617	100
TN-5	chr16	13811208	13822017	PGBD2	10809	100
TN-5	chr16	13829171	13884431	ENSCAFG00000043982	55260	100
TN-5	chr16	13991368	13992180	ENSCAFG00000046117	812	100
TN-5	chr16	14002066	14046567	ENSCAFG00000032319	44501	100
TN-5	chr16	22940216	22948582	THAP1	7656	92
TN-5	chr34	15829063	15914842	ATP11B	85779	100
TN-6	chr2	18368890	18403030	ENSCAFG00000045340	34140	100
TN-6	chr3	31290532	31291470	OR6C3I	938	100
TN-6	chr3	31300376	31301314	OR6C1	938	100
TN-6	chr3	31306813	31307487	ENSCAFG00000028828	674	100
TN-6	chr3	31362550	31363488	OR6C2D	938	100
TN-6	chr3	31382762	31383700	OR6C2G	938	100
TN-6	chr13	58911361	58932913	ENSCAFG00000029376	21552	100
TN-6	chr16	10449353	10450970	ENSCAFG00000024751	1617	100
TN-6	chr16	13811208	13822017	PGBD2	10809	100
TN-6	chr16	13829171	13884431	ENSCAFG00000043982	55260	100
TN-6	chr16	13947830	13948339	ENSCAFG00000049312	509	100
TN-6	chr16	13991368	13992180	ENSCAFG00000046117	812	100
TN-6	chr16	14002066	14046567	ENSCAFG00000032319	44501	100
TN-6	chr16	54054746	54056353	ENSCAFG00000049688	1607	100
TN-6	chr29	7185678	7187043	RAB28	1365	100
TN-6	chr36	10601280	10601561	ENSCAFG00000041552	281	100
TN-7	chr2	83901018	84132529	VPS13D	231394	100
TN-7	chr2	84120945	84121388	ENSCAFG00000045358	443	100
TN-7	chr9	11717163	11736890	CCDC47	19727	100
TN-8	chr1	18321021	18337430	NARS1	16409	100
TN-8	chr1	57162197	57169557	FAM162B	7360	100
TN-8	chr21	21136931	21160703	CLNS1A	23362	98
TN-8	chr21	27955995	27956945	OR52A18	950	100
TN-8	chr21	27968957	27969907	OR52A5C	950	100
TN-8	chr21	27987233	27988183	OR52A5	950	100
TN-8	chr21	28025759	28026709	OR52A24	950	100
TN-8	chr21	28033762	28034643	OR52AE1	881	100

TN-9	chr8	22369790	22375025	C8H14orf28	5235	100
TN-9	chr10	13357620	13425285	TBC1D15	67665	100
TN-9	chrX	121092870	121094885	ENSCAFG00000023624	2015	100
TN-10	chr13	2142876	2169944	RNF19A	27068	100
TN-10	chr16	54054746	54056353	ENSCAFG00000049688	1607	100
TN-10	chr18	40710931	40711863	OR4C146	932	100
TN-10	chr18	40723795	40724727	OR4C137	932	100
TN-10	chr18	40755753	40756667	OR4C138	914	100
TN-10	chr18	40775586	40794895	ENSCAFG00000044632	19309	100
TN-10	chr18	40828545	40829471	OR4C6	926	100
TN-10	chr22	16322199	16323306	ENSCAFG00000049473	1107	100
TN-10	chr23	38596643	38646401	U2SURP	49312	99
TN-10	chr28	29731950	29750229	TIAL1	18279	100
TN-10	chr37	7169192	7216590	BOLL	43306	91
TN-10	chrX	105685976	105686988	ENSCAFG00000041731	1012	100
TN-10	chrX	121092870	121094885	ENSCAFG00000023624	2015	100
TN-10	chrX	121173094	121174224	ZNF275	1130	100
TN-11	chr1	57162197	57169557	FAM162B	7360	100
TN-11	chr5	32878338	32888316	ALOX15B	9978	100
TN-11	chr10	13357620	13425285	TBC1D15	67665	100
TN-11	chr13	2142876	2169944	RNF19A	27068	100
TN-11	chr15	41036428	41037553	PMCH	1125	100
TN-11	chr27	5407906	5463809	TUBA1B	54078	97
TN-11	chr28	368513	406401	MAPK8	37888	100
TN-11	chr28	22227968	22241316	ENSCAFG00000030782	13348	100
TN-11	chr28	22285125	22336545	SHOC2	51420	100
TN-11	chr36	10601280	10601561	ENSCAFG00000041552	281	100
TN-12	chr2	18368890	18403030	ENSCAFG00000045340	34140	100
TN-12	chr8	22369790	22375025	C8H14orf28	5235	100
TN-12	chr28	368513	406401	MAPK8	37888	100
TN-12	chr38	70452	72976	BTG2	2524	100
TN-12	chr38	103230	112154	FMOD	8924	100
TN-13	chr1	121530203	121614909	URI1	81889	97
TN-14	chr12	39904603	40032194	PHIP	120240	94
TN-14	chr19	19855013	19935019	ENSCAFG00000012482	79006	99
TN-14	chr26	9423486	9426233	ENSCAFG00000015330	2747	100
TN-14	chr26	9446060	9449498	ENSCAFG00000008764	3438	100
TN-14	chr26	16537016	16546514	MLEC	9158	96
TN-14	chr26	16557742	16566257	UNC119B	8515	100
TN-14	chr26	16572947	16584194	ACADS	11247	100
TN-14	chr26	17170680	17214631	GIT2	43951	100
TN-14	chr26	17229123	17241270	TCHP	12147	100

TN-14	chr37	12086749	12140228	CYP20A1	53479	100
TN-15	chr5	82487597	82534561	TERB1	46964	100
TN-15	chr8	48110050	48116686	EIF2B2	6636	100
TN-15	chr8	48124105	48154102	MLH3	29997	100
TN-15	chr8	48155412	48169261	ACYP1	13849	100
TN-15	chr8	48170112	48215954	ZC2HC1C	45842	100
TN-15	chr8	48331026	48333467	FOS	2441	100
TN-15	chr8	53771252	53824298	SEL1L	53046	100
TN-15	chr12	39904603	40032194	PHIP	114833	90
TN-15	chr12	42671543	42760546	IBTK	89003	100
TN-15	chr12	42854129	42854720	ENSCAFG00000045685	591	100
TN-15	chr12	42871239	42875151	TPBG	3912	100
TN-15	chr12	44927542	44929104	ENSCAFG00000048629	1562	100
TN-15	chr12	65960066	66057035	SESN1	96969	100
TN-15	chr12	66833995	66897483	WASF1	62555	99
TN-15	chr12	66897412	66949366	CDC40	50974	98
TN-15	chr12	66958427	67104999	METTL24	146572	100
TN-15	chr12	67081329	67104397	DDO	23068	100
TN-15	chr24	19237962	19422553	ENSCAFG00000006763	177564	96
TN-15	chr36	10601280	10601561	ENSCAFG00000041552	281	100
TN-16	chr16	35473913	35499487	ERI1	25574	100
TN-16	chr18	49090689	49104051	MRPL21	13362	100
TN-17	chr8	22369790	22375025	C8H14orf28	5235	100
TN-17	chr16	6691254	6706207	TRPV6	14953	100
TN-17	chr16	9066970	9244526	HIPK2	172123	97
TN-17	chr16	9263450	9270531	CLEC2L	7081	100
TN-17	chr16	22784267	22901991	HOOK3	117443	100
TN-17	chr16	22900313	22927125	RNF170	26812	100
TN-17	chr16	22940216	22948582	THAP1	8366	100
TN-17	chr16	23604200	23724252	KAT6A	119552	100
TN-17	chr16	23766193	23964226	ANK1	197533	100
TN-17	chr16	23911972	24010152	GPAT4	97563	99
TN-17	chr16	23968783	23973803	ENSCAFG00000049702	5020	100
TN-17	chr27	904102	924084	ITGA5	19982	100
TN-17	chr27	928349	947186	ZNF385A	18837	100
TN-17	chr27	952886	954067	GPR84	1181	100
TN-17	chr27	964218	983010	ENSCAFG00000006536	18792	100
TN-17	chr27	1003816	1010264	NFE2	6448	100
TN-17	chr27	1015868	1019891	HNRNPA1	4023	100
TN-17	chr27	1039594	1049788	CBX5	10194	100
TN-17	chr34	15829063	15914842	ATP11B	78244	91
TN-18	chr7	57523595	57556005	RNF138	32410	100

TN-18	chr7	57574738	57608017	RNF125	33279	100
TN-18	chr14	737519	738328	ENSCAFG00000043502	809	100
TN-18	chr30	2980437	3146568	DPH6	155178	93
TN-18	chr30	37816681	37819334	ENSCAFG00000049897	2653	100
TN-18	chr33	280590	282779	CSNKA2IP	2189	100
TN-18	chr36	18024627	18077587	SP3	51815	98
TN-19	chr2	35346168	35346326	IGIP	158	99
TN-19	chr2	47991716	48063680	ZSWIM6	68357	95
TN-19	chr2	47995347	48058688	ENSCAFG00000047178	63341	100
TN-19	chr6	12061352	12065474	ENSCAFG00000030253	4122	100
TN-19	chr7	5962880	5963836	ENSCAFG00000014787	956	100
TN-19	chr9	33066726	33109787	HSF5	43061	100
TN-19	chr22	57005559	57006671	ABHD13	1112	100
TN-20	chr17	7104961	7122939	RRM2	16784	93
TN-20	chr17	21474914	21492460	ENSCAFG00000023355	16412	94
TN-20	chr17	21545673	21673157	ENSCAFG00000005160	127484	100
TN-20	chr17	21566827	21688006	ENSCAFG00000029636	121179	100
TN-20	chr17	21652664	21654586	ENSCAFG00000024084	1922	100
TN-20	chr30	15217722	15273953	SECISBP2L	56231	100
TN-22	chr5	42143474	42169517	COPS3	26043	100
TN-22	chr9	10277802	10280466	ENSCAFG00000042619	2664	100
TN-22	chr9	10282539	10283222	ENSCAFG00000015156	683	100
TN-22	chr9	10290616	10330701	ENSCAFG00000028642	40085	100
TN-22	chr9	11717163	11736890	CCDC47	19727	100
TN-22	chr9	18001439	18010441	ENSCAFG00000029973	8631	96
TN-22	chr9	18011787	18040219	ENSCAFG00000046940	28419	100
TN-22	chr9	18026718	18032842	ENSCAFG00000046006	6124	100
TN-22	chr9	18147118	18148665	ENSCAFG00000048685	1547	100
TN-22	chr12	15538356	15681011	CD2AP	142655	100
TN-22	chr13	58911361	58932913	ENSCAFG00000029376	21552	100
TN-22	chr14	737519	738328	ENSCAFG00000043502	809	100
TN-22	chr14	48873990	48880266	THAP5	6276	100
TN-22	chr14	48879914	48884361	DNAJB9	4447	100
TN-22	chr18	37392199	37393663	OR5A2B	1464	100
TN-22	chr23	3616259	3617315	ENSCAFG00000002132	1056	100
TN-22	chr24	5418977	5419132	ENSCAFG00000049664	155	99
TN-22	chr24	5439512	5499307	BFSP1	59795	100
TN-22	chr24	5556840	5560667	ENSCAFG00000047967	3827	100
TN-22	chr24	24594264	24614902	NFS1	20638	100
TN-22	chr24	24615197	24616582	ROMO1	1385	100
TN-22	chr24	24618890	24619203	ENSCAFG00000048937	313	100
TN-22	chr24	24619691	24648474	RBM39	26301	91

TN-22	chr25	18268445	18269479	ENSCAFG00000040982	1034	100
TN-22	chr25	18287899	18359553	PSPC1	71654	100
TN-22	chr26	6523070	6525123	ARL6IP4	2053	100
TN-22	chr26	6526304	6530286	OGFOD2	3982	100
TN-22	chr26	6544796	6576237	ABCB9	31441	100
TN-22	chr26	6595030	6620408	VPS37B	25377	100
TN-22	chr26	9423486	9426233	ENSCAFG00000015330	2734	99
TN-22	chr26	9446060	9449498	ENSCAFG00000008764	3438	100
TN-22	chr26	24278802	24296154	PATZ1	17352	100
TN-22	chr26	24327739	24350659	DRG1	22920	100
TN-22	chr26	24355826	24402827	EIF4ENIF1	47001	100
TN-22	chr38	2160233	2170560	NUCKS1	10327	100
TN-22	chr38	2198040	2203896	RAB29	5856	100
TN-22	chr38	21262789	21265887	APOA2	3098	100
TN-22	chr38	21268001	21270940	FCER1G	2939	100
TN-22	chr38	21271675	21285279	NDUFS2	13604	100
TN-22	chr38	21284330	21292437	ADAMTS4	8107	100
TN-22	chr38	21302003	21312958	B4GALT3	10955	100
TN-22	chr38	21306974	21311012	PPOX	4038	100
TN-22	chr38	21311859	21320106	USP21	8247	100
TN-22	chr38	21319054	21340211	UFC1	21157	100
TN-22	chr38	21342534	21351972	DEDD	9438	100
TN-22	chr38	21348486	21356260	NIT1	7774	100
TN-22	chr38	21355678	21367619	PFDN2	11941	100
TN-22	chr38	21366194	21370328	KLHDC9	4134	100
TN-22	chr38	21375556	21392632	NECTIN4	17076	100
TN-22	chr38	21395373	21412047	ARHGAP30	16674	100
TN-22	chr38	21413050	21418569	USF1	5519	100
TN-22	chr38	21419346	21421427	TSTD1	2081	100
TN-22	chr38	21892993	21936458	COPA	43465	100
TN-22	chr38	21939018	21944602	PEX19	5584	100
TN-23	chr7	13546312	13546996	ENSCAFG00000007327	684	100
TN-23	chr7	19832664	19966936	PLA2G4A	122945	92
TN-23	chr8	22369790	22375025	C8H14orf28	5235	100
TN-23	chr9	8971236	8976195	ENSCAFG00000045713	4959	100
TN-23	chr9	8979811	8980154	ENSCAFG00000029865	343	100
TN-23	chr14	737519	738328	ENSCAFG00000043502	809	100
TN-23	chr26	31235813	31238461	ENSCAFG00000049465	2648	100
TN-24	chr1	112562437	112564498	ERICH4	2005	97
TN-24	chr1	112914936	112917332	ENSCAFG00000042954	2396	100
TN-24	chr2	36378640	36381614	PCDHGB5	2974	100
TN-24	chr6	12061352	12065474	ENSCAFG00000030253	4122	100

TN-24	chr11	45181602	45186893	LRRC19	5291	100
TN-24	chr21	27955995	27956945	OR52A18	950	100
TN-24	chr21	27968957	27969907	OR52A5C	950	100
TN-24	chr21	27987233	27988183	OR52A5	950	100
TN-24	chr21	28025759	28026709	OR52A24	950	100
TN-24	chr21	28033762	28034643	OR52AE1	881	100
TN-24	chrX	105685976	105686988	ENSCAFG00000041731	1012	100
TN-24	chrX	105735708	105737321	ENSCAFG00000047366	1613	100
TN-24	chrX	105744248	105754765	ENSCAFG00000046904	10517	100
TN-25	chr2	35761626	35762600	SLC35A4	974	100
TN-25	chr2	67789035	67835436	NETO2	46401	100
TN-25	chr8	44002032	44002277	ENSCAFG00000016617	245	100
TN-25	chr11	688383	690491	ENSCAFG00000045515	2108	100
TN-25	chr11	739729	741951	ENSCAFG00000042532	2222	100
TN-25	chr11	35079886	35131372	ZDHHC21	51486	100
TN-25	chr12	68499525	68514648	TUBE1	15123	100
TN-25	chr18	13126050	13127351	GPR22	1301	100
TN-25	chr21	27955995	27956945	OR52A18	950	100
TN-25	chr21	27968957	27969907	OR52A5C	950	100
TN-25	chr21	27987233	27988183	OR52A5	950	100
TN-25	chr21	28025759	28026709	OR52A24	950	100
TN-25	chr21	28033762	28034643	OR52AE1	881	100
TN-25	chr27	1770044	1770457	PRR13	388	94
TN-25	chrX	105685976	105686988	ENSCAFG00000041731	1012	100
TN-26	chr2	19204529	19234522	TMEM236	29993	100
TN-26	chr2	54317534	54350742	CDK7	33208	100
TN-26	chr2	54335419	54336721	ENSCAFG00000007757	1302	100
TN-26	chr2	84120945	84121388	ENSCAFG00000045358	443	100
TN-26	chr5	42143474	42169517	COPS3	25976	100
TN-26	chr5	82487597	82534561	TERB1	46964	100
TN-26	chr9	8971236	8976195	ENSCAFG00000045713	4959	100
TN-26	chr9	8979811	8980154	ENSCAFG00000029865	343	100
TN-26	chr9	8990441	8993428	ENSCAFG00000049561	2987	100
TN-26	chr9	10350542	10373321	ENSCAFG00000032099	22779	100
TN-26	chr9	18001439	18010441	ENSCAFG00000029973	9002	100
TN-26	chr9	18011787	18040219	ENSCAFG00000046940	28432	100
TN-26	chr9	18026718	18032842	ENSCAFG00000046006	6124	100
TN-26	chr9	18147118	18148665	ENSCAFG00000048685	1547	100
TN-26	chr9	22501070	22527843	MED24	24451	91
TN-26	chr10	15843975	15888355	CAPS2	44380	100
TN-26	chr10	15901517	15915819	ENSCAFG00000023219	14302	100
TN-26	chr10	15924513	15939282	GLIPR1L2	13929	94

TN-26	chr11	22385257	22410052	PPP2CA	24795	100
TN-26	chr28	368513	406401	MAPK8	37888	100
TN-26	chr33	19724122	19724649	ENSCAFG00000049619	527	100
TN-26	chr35	24092007	24124371	ENSCAFG00000024295	29680	92
TN-26	chr35	24121361	24121672	H4C4	311	100
TN-27	chr6	39048278	39076035	MEIOB	27757	100
TN-27	chr6	56679039	56723652	C6H1orf146	44613	100
TN-27	chr9	4743539	4752270	WBP2	8634	99
TN-27	chr9	10290616	10330701	ENSCAFG00000028642	40085	100
TN-27	chr9	17977636	17998135	ENSCAFG00000031375	20499	100
TN-27	chr14	7724780	7742795	IRF5	17505	97
TN-27	chr14	7751351	7780558	KCP	29207	100
TN-27	chr14	7784220	7786284	ATP6V1FNB	2064	100
TN-27	chr14	7786844	7789548	ATP6V1F	2704	100
TN-27	chr14	7793449	7819940	FLNC	26491	100
TN-27	chr14	40267528	40269681	HOXA1	2153	100
TN-27	chr14	40274469	40276239	HOXA2	1770	100
TN-27	chr14	40280861	40296147	HOXA3	15286	100
TN-27	chr14	40302816	40304321	HOXA4	1505	100
TN-27	chr14	40315639	40317768	HOXA5	2129	100
TN-27	chr14	40319706	40321800	HOXA6	2094	100
TN-27	chr14	40329127	40330842	HOXA7	1715	100
TN-27	chr14	40337999	40339923	HOXA9	1924	100
TN-27	chr14	40346462	40354396	HOXA10	7934	100
TN-27	chr14	43163571	43227103	NOD1	63532	100
TN-27	chr14	43245102	43252923	GGCT	7821	100
TN-27	chr30	20795966	20809490	C15orf65	13524	100
TN-27	chr30	20815775	20871220	DNAAF4	55445	100
TN-27	chr30	23606517	23731823	ADAM10	125306	100
TN-27	chr30	23753681	23821254	MINDY2	67573	100
TN-27	chr38	21451952	21454959	F11R	3007	100
TN-27	chr38	21495720	21517043	CD244	21323	100
TN-27	chr38	21523637	21554579	LY9	30942	100
TN-27	chr38	21566748	21628219	CD48	61471	100
TN-27	chr38	21581439	21596348	SLAMF7	14909	100
TN-27	chr38	21653487	21685408	SLAMF1	31921	100
TN-27	chr38	21685868	21892097	NCSTN	206028	100
TN-27	chr38	21702494	21759668	CD84	57174	100
TN-27	chr38	21742301	21764494	SLAMF6	22193	100
TN-27	chr38	21892993	21936458	COPA	43465	100
TN-27	chr38	21939018	21944602	PEX19	5584	100
TN-27	chr38	21959254	21999677	DCAF8	40423	100

TN-28	chr5	42143474	42169517	COPS3	25976	100
TN-28	chr33	24151700	24186512	GTF2E1	34812	100
TN-29	chr7	13546312	13546996	ENSCAFG00000007327	684	100
TN-29	chr18	45142586	45159255	ACCS	16669	100
TN-29	chr18	45168245	45173107	PIDD1	4862	100
TN-29	chr18	45176610	45178278	ENSCAFG00000009523	1668	100
TN-29	chr18	45182566	45187268	PNPLA2	4702	100
TN-29	chr18	45190250	45194038	CRACR2B	3788	100
TN-29	chr18	45196237	45200081	CD151	3844	100
TN-29	chr18	45203620	45224674	TSPAN4	21054	100
TN-29	chr18	45226865	45256750	CHID1	29869	100
TN-29	chr18	45253772	45358018	AP2A2	97524	94
TN-29	chr18	45360028	45373325	ENSCAFG00000032054	13297	100
TN-29	chr18	45416331	45438238	ENSCAFG00000044092	21907	100
TN-29	chr18	46206619	46207997	ENSCAFG00000043534	1378	100
TN-29	chr18	46294019	46311982	IGF2	17963	100
TN-29	chr18	46311865	46313734	ENSCAFG00000046194	1869	100
TN-29	chr18	46324041	46325122	INS	1081	100
TN-29	chr18	46327137	46335602	TH	8465	100
TN-29	chr18	46395391	46395975	ASCL2	584	100
TN-29	chr18	46422259	46437272	TSPAN32	15013	100
TN-29	chr18	49090689	49104051	MRPL21	13362	100
TN-29	chr18	49136691	49190117	CPT1A	50800	95
TN-29	chr18	49191211	49230576	TESMIN	39365	100
TN-29	chr18	49216889	49217257	ENSCAFG00000010770	368	100
TN-29	chr18	49240437	49246950	GAL	6513	100
TN-29	chr18	49289044	49385656	PPP6R3	94018	97
TN-29	chr18	49439483	49516396	LRP5	76913	100
TN-29	chr18	54659406	54670623	FADS1	10290	92
TN-29	chr29	7185678	7187043	RAB28	1365	100
TN-29	chr35	23840934	23853385	SLC17A2	12451	100
TN-29	chr35	23883486	23884110	ENSCAFG00000010760	624	100
TN-29	chr35	23893537	23909249	ENSCAFG00000025396	15712	100
TN-29	chr35	23926287	23939466	TRIM38	13179	100
TN-29	chr35	23977201	23977611	H3C1	410	100
TN-29	chr35	23978521	23978832	H4C1	311	100
TN-29	chr35	24001179	24012425	ENSCAFG00000046600	11246	100
TN-29	chr35	24001900	24002292	H2AC5	392	100
TN-29	chr35	24003167	24003577	H3C3	410	100
TN-29	chr35	24031387	24042413	HFE	10131	92
TN-29	chr35	24377632	24378840	ZNF322	1208	100
TN-29	chr35	24556015	24556326	H4C9	311	100

TN-29	chr35	24557784	24558164	H2BC12	380	100
TN-30	chr2	84120945	84121388	ENSCAFG00000045358	443	100
TN-30	chr7	2234504	2262622	ENSCAFG00000030161	28118	100
TN-30	chr7	13546312	13546996	ENSCAFG00000007327	618	90
TN-30	chr13	2142876	2169944	RNF19A	27068	100
TN-30	chr15	36023519	36076381	CDK17	52862	100
TN-30	chr15	36103141	36103561	ENSCAFG00000046308	420	100
TN-30	chr26	28931806	28934982	ENSCAFG00000046712	3176	100
TN-30	chr29	7185678	7187043	RAB28	1365	100
TN-31	chr9	26183852	26199927	COL1A1	16075	100
TN-31	chr9	26256260	26261056	TMEM92	4796	100
TN-31	chr9	26316774	26329511	XYLT2	12506	98
TN-31	chr9	35166353	35191932	TBX4	25579	100
TN-31	chr9	35211808	35212119	ENSCAFG00000017742	311	100
TN-31	chr9	35235649	35245736	TBX2	10087	100
TN-31	chr9	47368544	47401362	TRPV3	32818	100
TN-31	chr9	47404972	47431926	TRPV1	26954	100
TN-31	chr9	47446342	47474107	ENSCAFG00000019344	27765	100
TN-31	chr9	47473901	47490252	CTNS	16351	100
TN-31	chr9	47493069	47497203	TAX1BP3	4134	100
TN-31	chr9	47497719	47498048	EMC6	329	100
TN-31	chr9	47502598	47516636	ENSCAFG00000023152	14038	100
TN-31	chr9	47533305	47596209	ITGAE	62904	100
TN-31	chr9	47540446	47542791	HASPIN	2345	100
TN-31	chr13	21377316	21379832	ZHX2	2516	100
TN-31	chr22	61283309	61312283	CDC16	28974	100
TN-31	chr22	61320451	61345839	UPF3A	25388	100
TN-31	chr22	61368049	61378111	CHAMP1	10062	100
TN-31	chr25	34710934	34721006	ENSCAFG00000042909	9411	93
TN-31	chr25	34725226	34838994	PPP3CC	113768	100
TN-31	chr38	523481	720195	SOX13	183874	93
TN-32	chr1	112914936	112917332	ENSCAFG00000042954	2396	100
TN-32	chr6	54742111	54817235	ABCD3	74824	100
TN-32	chr16	9413359	9418791	FMC1	5432	100
TN-32	chr16	22940216	22948582	THAP1	8256	99
TN-32	chr18	40710931	40711863	OR4C146	932	100
TN-32	chr18	40723795	40724727	OR4C137	932	100
TN-32	chr18	40755753	40756667	OR4C138	914	100
TN-32	chr18	40775586	40794895	ENSCAFG00000044632	19309	100
TN-32	chr29	7185678	7187043	RAB28	1365	100
TN-32	chr36	18024627	18077587	SP3	52960	100
TN-33	chr7	57523595	57556005	RNF138	32410	100

TN-33	chr18	12703341	12734479	DLD	31098	100
TN-33	chr18	54335036	54338105	ENSCAFG00000029389	3069	100
TN-33	chr20	54224789	54225841	FUT5	1052	100
TN-33	chr37	12086749	12140228	CYP20A1	53479	100
TN-34	chr5	32878338	32888316	ALOX15B	9689	97
TN-34	chr9	10277802	10280466	ENSCAFG00000042619	2664	100
TN-34	chr9	10282539	10283222	ENSCAFG00000015156	683	100
TN-34	chr9	10290616	10330701	ENSCAFG00000028642	40085	100
TN-34	chr26	9446060	9449498	ENSCAFG00000008764	3438	100
TN-34	chr36	10601280	10601561	ENSCAFG00000041552	281	100
TN-35	chr11	35079886	35131372	ZDHHC21	51486	100
TN-35	chr18	40710931	40711863	OR4C146	932	100
TN-35	chr18	40723795	40724727	OR4C137	932	100
TN-35	chr18	40755753	40756667	OR4C138	914	100
TN-35	chr18	40775586	40794895	ENSCAFG00000044632	19309	100
TN-35	chr18	40828545	40829471	OR4C6	926	100
TN-36	chr5	82487597	82534561	TERB1	46964	100
TN-36	chr16	10449353	10450970	ENSCAFG00000024751	1617	100
TN-36	chr18	40710931	40711863	OR4C146	932	100
TN-36	chr18	40723795	40724727	OR4C137	932	100
TN-36	chr18	40755753	40756667	OR4C138	914	100
TN-36	chr18	40775586	40794895	ENSCAFG00000044632	19309	100
TN-36	chr18	40828545	40829471	OR4C6	926	100
TN-36	chr30	25244644	25308718	ICE2	64074	100
TN-36	chr36	10601280	10601561	ENSCAFG00000041552	281	100
TN-37	chr2	18368890	18403030	ENSCAFG00000045340	34140	100
TN-37	chr16	54054746	54056353	ENSCAFG00000049688	1607	100
TN-37	chr18	49090689	49104051	MRPL21	13362	100
TN-38	chr11	60715165	60757351	NIPSNAP3B	39090	93
TN-38	chr17	39326023	39403026	ENSCAFG00000031333	77003	100
TN-38	chr17	56680301	56698419	HMGCS2	18118	100
TN-38	chr17	56728398	56772642	ENSCAFG00000029705	44244	100
TN-39	chr5	32878338	32888316	ALOX15B	9689	97
TN-39	chr8	22369790	22375025	C8H14orf28	5149	98
TN-39	chr9	10350542	10373321	ENSCAFG00000032099	22779	100
TN-39	chr9	10389871	10393698	ENSCAFG00000048980	3827	100
TN-39	chr16	10449353	10450970	ENSCAFG00000024751	1617	100
TN-39	chr16	13811208	13822017	PGBD2	10809	100
TN-39	chr16	13829171	13884431	ENSCAFG00000043982	55260	100
TN-39	chr16	13991368	13992180	ENSCAFG00000046117	812	100
TN-39	chr16	14002066	14046567	ENSCAFG00000032319	44501	100
TN-40	chr16	41361436	41401007	ENSCAFG00000007135	37454	95

TN-40	chr16	54054746	54056353	ENSCAFG00000049688	1607	100
TN-41	chr3	56689469	56701927	TLNRD1	12458	100
TN-41	chr11	45181602	45186893	LRRC19	5291	100
TN-41	chr11	50675525	50802940	UBAP2	122531	96
TN-41	chr15	41036428	41037553	PMCH	1125	100
TN-41	chr17	56680301	56698419	HMGCS2	18118	100
TN-41	chr17	56728398	56772642	ENSCAFG00000029705	44244	100
TN-41	chr36	10601280	10601561	ENSCAFG00000041552	281	100
TN-42	chr8	2356960	2358745	ENSCAFG00000031023	1785	100
TN-42	chr8	2365535	2366207	ENSCAFG00000043621	672	100
TN-42	chr8	2370648	2371741	ENSCAFG00000043519	1093	100
TN-42	chr8	2372687	2375185	ENSCAFG00000049498	2498	100
TN-42	chr8	2406019	2408341	ENSCAFG00000046168	2322	100
TN-42	chr8	2411890	2412979	ENSCAFG00000047752	1089	100
TN-42	chr8	2418492	2418979	ENSCAFG00000014977	487	100
TN-42	chr8	2420714	2423384	ENSCAFG00000011085	2420	91
TN-42	chr16	41361436	41401007	ENSCAFG00000007135	39571	100
TN-42	chr20	57891413	57898863	RNF126	7450	100
TN-42	chr23	3616259	3617315	ENSCAFG00000002132	1056	100
TN-42	chr23	3617664	3619355	ENSCAFG00000029974	1691	100
TN-42	chr25	50731528	50738333	ENSCAFG00000030158	6805	100
TN-42	chr25	50771221	50945936	ENSCAFG00000042242	173500	99
TN-42	chr25	50839161	50841227	ENSCAFG00000047776	2066	100
TN-42	chr25	50858698	50944964	KIF1A	85641	99
TN-42	chr25	50967553	50975880	ENSCAFG00000029684	8327	100
TN-42	chr25	51004465	51038700	CROCC2	33104	97
TN-42	chr26	20101737	20122482	ASPHD2	20745	100
TN-43	chr12	2221137	2227600	DLA-DQA1	6463	100
TN-43	chr12	2244820	2251662	ENSCAFG00000000814	6842	100
TN-43	chr17	21474914	21492460	ENSCAFG00000023355	17546	100
TN-43	chr17	21545673	21673157	ENSCAFG00000005160	127484	100
TN-43	chr17	21566827	21688006	ENSCAFG00000029636	121179	100
TN-43	chr17	21652664	21654586	ENSCAFG00000024084	1922	100
TN-44	chr18	49090689	49104051	MRPL21	13362	100
TN-44	chr18	54335036	54338105	ENSCAFG00000029389	3069	100
TN-45	chr1	101855440	101877099	NLRP8	21659	100
TN-45	chr1	101896523	101921918	NLRP13	25395	100
TN-45	chr2	18368890	18403030	ENSCAFG00000045340	34140	100
TN-45	chr17	21474914	21492460	ENSCAFG00000023355	17546	100
TN-45	chr17	21545673	21673157	ENSCAFG00000005160	127484	100
TN-45	chr17	21566827	21688006	ENSCAFG00000029636	121179	100
TN-45	chr17	21652664	21654586	ENSCAFG00000024084	1922	100

TN-45	chr26	31235813	31238461	ENSCAFG00000049465	2648	100
TN-45	chrX	1032368	1038633	ENSCAFG00000030459	6265	100
TN-46	chr7	13546312	13546996	ENSCAFG00000007327	684	100
TN-46	chr7	80937643	80938650	ENSCAFG00000014968	1007	100
TN-46	chr11	35079886	35131372	ZDHHC21	51486	100
TN-46	chr18	49090689	49104051	MRPL21	13362	100
TN-46	chr23	30475978	30481632	CDV3	5574	99
TN-46	chr23	43870939	43918436	HLTF	46031	97
TN-46	chr37	12086749	12140228	CYP20A1	53479	100
TN-47	chr5	32711305	32712108	TMEM88	803	100
TN-47	chr7	2234504	2262622	ENSCAFG00000030161	28118	100
TN-47	chr7	13546312	13546996	ENSCAFG00000007327	684	100
TN-47	chr8	44002032	44002277	ENSCAFG00000016617	245	100
TN-47	chr16	10449353	10450970	ENSCAFG00000024751	1617	100
TN-47	chr25	19597518	19618180	CBR4	20246	98
TN-47	chr25	19599007	19599201	ENSCAFG00000042558	194	99
TN-47	chr28	29731950	29750229	TIAL1	18279	100
TN-47	chr31	38203732	38249207	ENSCAFG00000029897	45475	100
TN-47	chr34	30284858	30373780	SI	88922	100
TN-47	chr37	7169192	7216590	BOLL	43306	91
TN-47	chr37	12086749	12140228	CYP20A1	53479	100
TN-48	chr17	39326023	39403026	ENSCAFG00000031333	77003	100
TN-48	chr17	56680301	56698419	HMGCS2	18118	100
TN-48	chr17	56728398	56772642	ENSCAFG00000029705	44244	100
TN-48	chr36	10601280	10601561	ENSCAFG00000041552	281	100
TN-49	chr5	32878338	32888316	ALOX15B	9689	97
TN-49	chr16	22940216	22948582	THAP1	8256	99
TN-50	chr5	41965164	41966817	RASD1	1653	100
TN-50	chr16	10449353	10450970	ENSCAFG00000024751	1617	100
TN-50	chr20	55824410	55828980	TBXA2R	4570	100
TN-50	chr21	27955995	27956945	OR52A18	950	100
TN-50	chr21	27968957	27969907	OR52A5C	950	100
TN-50	chr21	27987233	27988183	OR52A5	950	100
TN-50	chr21	28025759	28026709	OR52A24	950	100
TN-50	chr21	28033762	28034643	OR52AE1	881	100
TN-51	chr4	43215582	43217124	FBLL1	1542	100
TN-51	chr4	68617594	68648929	TTC33	31335	100
TN-51	chr5	47525365	47532899	ANGPTL3	7140	95
TN-51	chr16	10449353	10450970	ENSCAFG00000024751	1617	100
TN-51	chr16	13811208	13822017	PGBD2	10809	100
TN-51	chr16	13829171	13884431	ENSCAFG00000043982	55260	100
TN-51	chr16	13991368	13992180	ENSCAFG00000046117	812	100

TN-51	chr16	14002066	14046567	ENSCAFG00000032319	44501	100
TN-51	chr17	57385047	57446097	ENSCAFG00000028490	61050	100
TN-51	chr25	50967553	50975880	ENSCAFG00000029684	8327	100
TN-51	chr25	50982758	50989100	ENSCAFG00000012788	6342	100
TN-51	chr27	5407906	5463809	TUBA1B	53487	96
TN-51	chr36	10601280	10601561	ENSCAFG00000041552	281	100
TN-51	chrX	105685976	105686988	ENSCAFG00000041731	1012	100
TN-52	chr1	106019171	106022517	KLK15	3346	100
TN-52	chr8	2273563	2274464	ENSCAFG00000048830	901	100
TN-52	chr8	2282856	2283504	ENSCAFG00000029069	648	100
TN-52	chr8	2289879	2290968	ENSCAFG00000048140	1089	100
TN-52	chr8	2295396	2380876	ENSCAFG00000045242	85480	100
TN-52	chr8	2304639	2305537	ENSCAFG00000014973	898	100
TN-52	chr8	2311539	2312292	ENSCAFG00000024337	753	100
TN-52	chr8	2323009	2323657	ENSCAFG00000028548	648	100
TN-52	chr8	2356960	2358745	ENSCAFG00000031023	1785	100
TN-52	chr8	2365535	2366207	ENSCAFG00000043621	672	100
TN-52	chr8	2370648	2371741	ENSCAFG00000043519	1093	100
TN-52	chr8	2372687	2375185	ENSCAFG00000049498	2498	100
TN-52	chr8	2406019	2408341	ENSCAFG00000046168	2322	100
TN-52	chr8	2411890	2412979	ENSCAFG00000047752	1089	100
TN-52	chr8	2418492	2418979	ENSCAFG00000014977	487	100
TN-52	chr10	11262265	11292832	CPSF6	27895	91
TN-52	chr10	17315015	17318512	ZBED4	3497	100
TN-52	chr12	72459041	72479124	TBP	20083	100
TN-52	chr12	72482886	72489437	PDCD2	6505	99
TN-52	chr14	38095716	38213418	MPP6	110545	94
TN-52	chr17	61096181	61097412	ENSCAFG00000030077	1231	100
TN-52	chr18	13126050	13127351	GPR22	1301	100
TN-52	chr27	22257941	22293369	KRAS	32830	93
TN-52	chr34	30284858	30373780	SI	88717	100
TN-52	chr34	30447659	30450598	SLITRK3	2939	100
TN-52	chr36	7749191	7778968	GCA	27985	94
TN-52	chrX	61980167	61982446	ENSCAFG00000046786	2279	100
TN-53	chr3	31290532	31291470	OR6C3I	938	100
TN-53	chr3	31300376	31301314	OR6C1	938	100
TN-53	chr3	31306813	31307487	ENSCAFG00000028828	674	100
TN-53	chr3	31362550	31363488	OR6C2D	938	100
TN-53	chr3	31382762	31383700	OR6C2G	938	100
TN-53	chr5	32878338	32888316	ALOX15B	9978	100
TN-53	chr8	2311539	2312292	ENSCAFG00000024337	753	100
TN-53	chr8	2323009	2323657	ENSCAFG00000028548	648	100

TN-53	chr8	2356960	2358745	ENSCAFG00000031023	1785	100
TN-53	chr8	2365535	2366207	ENSCAFG00000043621	672	100
TN-53	chr8	2370648	2371741	ENSCAFG00000043519	1093	100
TN-53	chr8	2372687	2375185	ENSCAFG00000049498	2498	100
TN-53	chr10	36330617	36332123	ENSCAFG00000041082	1484	98
TN-53	chr16	10449353	10450970	ENSCAFG00000024751	1617	100
TN-53	chr16	13811208	13822017	PGBD2	10809	100
TN-53	chr16	13829171	13884431	ENSCAFG00000043982	55260	100
TN-53	chr16	13947830	13948339	ENSCAFG00000049312	509	100
TN-53	chr16	13991368	13992180	ENSCAFG00000046117	812	100
TN-53	chr16	14002066	14046567	ENSCAFG00000032319	44501	100
TN-53	chr16	41361436	41401007	ENSCAFG00000007135	39571	100
TN-53	chr29	7185678	7187043	RAB28	1365	100
TN-54	chr4	66739148	66769075	ZNF131	29402	98
TN-54	chr10	11262265	11292832	CPSF6	30084	98
TN-54	chr14	737519	738328	ENSCAFG00000043502	809	100
TN-54	chr17	21474914	21492460	ENSCAFG00000023355	17536	100
TN-54	chr17	21545673	21673157	ENSCAFG00000005160	127484	100
TN-54	chr17	21566827	21688006	ENSCAFG00000029636	121179	100
TN-54	chr17	21652664	21654586	ENSCAFG00000024084	1922	100
TN-54	chr17	25082291	25085854	ENSCAFG00000049875	3563	100
TN-54	chr25	37733229	37800886	CUL3	67657	100
TN-54	chr25	51581546	51597774	GAL3ST2	16228	100
TN-54	chr25	51600491	51602507	ENSCAFG00000045576	2016	100
TN-54	chr25	51608173	51618723	PDCD1	10550	100
TN-54	chr25	51624447	51628668	ENSCAFG00000013188	4221	100
TN-54	chr33	13319266	13363580	CD47	44150	100
TN-54	chr33	24151700	24186512	GTF2E1	34812	100
TN-55	chr8	35737058	35748893	SIX4	11710	99
TN-55	chr8	72416637	72417384	PLD4	747	100
TN-55	chr10	11262265	11292832	CPSF6	27895	91
TN-55	chr19	19855013	19935019	ENSCAFG00000012482	79006	99
TN-55	chr20	57891413	57898863	RNF126	7450	100
TN-55	chr20	57900416	57903061	FGF22	2645	100
TN-55	chr33	24151700	24186512	GTF2E1	34681	100

Supplementary Table S3 a) Summary of genetic amplifications in the dataset overlapping >90% of the gene transcript. The canine reference assembly CanFam3.1 was used for the alignment and annotation.

Sample	Chromosome	Gene_Start	Gene_Stop	Gene_Name	Length_Deleted	%_of_Gene_Deleted
TN-1	chr30	751419	752048	ENSCAFG00000047951	629	100
TN-2	chr3	53358045	53365423	FURIN	7378	100
TN-2	chr5	81792155	81804152	CARMIL2	11997	100
TN-2	chr7	497394	502952	SYT2	5558	100
TN-2	chr12	3533102	3538533	HMGA1	5431	100
TN-2	chr20	48150498	48157191	GIPC1	6693	100
TN-2	chr26	29528366	29568522	GP1BB	38276	95
TN-2	chr26	29569586	29573842	SEPTIN5	4256	100
TN-2	chr30	751419	752048	ENSCAFG00000047951	629	100
TN-2	chr36	6989488	6997900	TBR1	8412	100
TN-2	chrX	41818950	41825811	WDR13	6861	100
TN-2	chrX	41882875	41890058	WAS	7183	100
TN-2	chrX	41893045	41906403	SUV39H1	13358	100
TN-2	chrX	41967812	41979243	GLOD5	11431	100
TN-2	chrX	41971582	41998303	GATA1	26721	100
TN-2	chrX	42004591	42024427	HDAC6	19836	100
TN-2	chrX	120910842	120912251	PNMA6A	1409	100
TN-3	chr8	3359875	3367942	CDH24	7398	92
TN-3	chr26	18882977	18884310	ENSCAFG00000011396	1333	100
TN-3	chr35	3525780	3540295	PSMG4	14515	100
TN-3	chr35	3545097	3692422	SLC22A23	147325	100
TN-4	chr5	41663773	41686776	SREBF1	23003	100
TN-4	chr7	731931	834893	LGR6	100735	98
TN-4	chr7	837273	853840	ENSCAFG00000010570	16567	100
TN-4	chr7	859214	869183	PTPN7	9969	100
TN-4	chr7	859585	963788	ELF3	104203	100
TN-4	chr7	883279	887811	GPR37L1	4532	100
TN-4	chr12	2663515	2669917	SLC39A7	6402	100
TN-4	chr19	3483542	3578870	ELF2	95328	100
TN-4	chr19	3588695	3609307	NOCT	20612	100
TN-4	chr27	5509874	5512909	RHEBL1	3035	100
TN-4	chr27	5521804	5553490	KMT2D	31686	100
TN-4	chr27	5609735	5629641	ENSCAFG00000047376	19906	100
TN-4	chr27	5636627	5639509	FKBP11	2882	100
TN-4	chr27	5639893	5650109	CCDC65	10216	100
TN-4	chr27	5679658	5687428	RND1	7770	100
TN-4	chr28	11517860	11545337	PYROXD2	27477	100
TN-4	chr28	11547741	11571613	HPS1	23872	100

TN-4	chr36	2547421	2552780	NR4A2	5359	100
TN-5	chr5	32696211	32710666	KDM6B	14455	100
TN-5	chr5	32711305	32712108	TMEM88	803	100
TN-5	chr5	32712805	32713600	NAA38	795	100
TN-5	chr5	32714187	32715680	CYB5D1	1493	100
TN-5	chr5	67001022	67180979	GSE1	167448	93
TN-5	chr8	4127488	4128794	FITM1	1306	100
TN-5	chr8	4131345	4134006	PSME1	2661	100
TN-5	chr8	4134020	4136290	EMC9	2270	100
TN-5	chr8	4137247	4140706	PSME2	3459	100
TN-5	chr10	1876536	1898348	CTDSP2	21812	100
TN-5	chr10	62830187	62837172	OTX1	6985	100
TN-5	chr12	2657792	2663706	RXRB	5914	100
TN-5	chr12	2663515	2669917	SLC39A7	6402	100
TN-5	chr12	12660008	12664940	HSP90AB1	4932	100
TN-5	chr12	12664871	12668484	SLC35B2	3613	100
TN-5	chr12	12669187	12673732	ENSCAFG00000001985	4545	100
TN-5	chr16	6287910	6304090	EPHA1	16180	100
TN-5	chr16	6304477	6311825	ZYX	7348	100
TN-5	chr16	15111116	15126617	SLC4A2	15501	100
TN-5	chr16	15124704	15130917	FASTK	6213	100
TN-5	chr16	15131354	15133716	TMUB1	2362	100
TN-5	chr27	1234689	1236137	HOXC6	1448	100
TN-5	chr27	1252730	1254876	HOXC8	2146	100
TN-5	chr27	1287380	1289372	HOXC11	1992	100
TN-5	chr27	1715158	1726885	TARBP2	11727	100
TN-5	chr27	25779162	25782134	ENSCAFG00000016278	2972	100
TN-5	chr27	25814516	25816336	ENSCAFG00000016289	1820	100
TN-5	chr34	20135390	20135503	ENSCAFG00000045746	113	99
TN-5	chr37	24952902	24958137	TMBIM1	4931	94
TN-5	chr37	25050267	25056113	CTDSP1	5846	100
TN-5	chr37	26008035	26009928	SPEGNB	1893	100
TN-5	chr37	26010638	26017787	GMPPA	7149	100
TN-5	chr37	26016605	26017193	ENSCAFG00000048574	588	100
TN-5	chr37	26024947	26048077	ASIC4	23130	100
TN-5	chr37	26025993	26052806	CHPF	26813	100
TN-5	chr37	26052168	26058810	TMEM198	6642	100
TN-6	chr2	81220431	81224243	MFAP2	3812	100
TN-6	chr7	44820	73051	ADORA1	28231	100
TN-6	chr7	112034	113892	MYOG	1858	100
TN-6	chr12	16856162	16858711	ENSCAFG00000002106	2448	96
TN-6	chr29	41768470	41769408	OR6C75C	938	100

TN-6	chrX	41818950	41825811	WDR13	6861	100
TN-6	chrX	41882875	41890058	WAS	7183	100
TN-6	chrX	41893045	41906403	SUV39H1	13358	100
TN-6	chrX	41967812	41979243	GLOD5	11431	100
TN-6	chrX	41971582	41998303	GATA1	26721	100
TN-6	chrX	42004591	42024427	HDAC6	19836	100
TN-8	chr5	9673809	9679799	NRGN	5873	98
TN-8	chrX	41818950	41825811	WDR13	6861	100
TN-8	chrX	41882875	41890058	WAS	7183	100
TN-8	chrX	41893045	41906403	SUV39H1	13358	100
TN-8	chrX	41967812	41979243	GLOD5	11431	100
TN-8	chrX	41971582	41998303	GATA1	26721	100
TN-8	chrX	42004591	42024427	HDAC6	19836	100
TN-9	chrX	41806176	41809481	RBM3	3305	100
TN-9	chrX	41818950	41825811	WDR13	6861	100
TN-9	chrX	41882875	41890058	WAS	7183	100
TN-9	chrX	41893045	41906403	SUV39H1	13358	100
TN-9	chrX	41967812	41979243	GLOD5	11431	100
TN-9	chrX	41971582	41998303	GATA1	26721	100
TN-9	chrX	42004591	42024427	HDAC6	19836	100
TN-9	chrX	120910842	120912251	PNMA6A	1409	100
TN-9	chrX	121013351	121015333	ENSCAFG00000019182	1982	100
TN-9	chrX	121031035	121042030	ENSCAFG00000023819	10995	100
TN-10	chr18	49090689	49104051	MRPL21	13362	100
TN-10	chrX	120910842	120912251	PNMA6A	1409	100
TN-11	chr1	104670684	104688413	ENSCAFG00000029058	17716	100
TN-11	chr1	112117141	112124077	ERF	6936	100
TN-11	chr1	118907184	118923215	SLC7A10	16031	100
TN-11	chr1	118926861	118929769	LRP3	2908	100
TN-11	chr2	81196463	81215689	ATP13A2	19226	100
TN-11	chr2	81220431	81224243	MFAP2	3812	100
TN-11	chr2	81860234	81863494	SLC25A34	3260	100
TN-11	chr5	30526720	30539483	MYBBP1A	12763	100
TN-11	chr5	30540450	30543773	GGT6	3323	100
TN-11	chr5	30754963	30819443	PITPNM3	64480	100
TN-11	chr5	30820867	30825300	PIMREG	4433	100
TN-11	chr5	36958264	36978606	ELAC2	20342	100
TN-11	chr5	56239701	56272745	AGRN	33044	100
TN-11	chr5	56283491	56287251	ENSCAFG00000028544	3760	100
TN-11	chr5	56503720	56506898	TAS1R3	3178	100
TN-11	chr5	56507739	56519491	DVL1	11752	100
TN-11	chr5	64563192	64565445	APRT	2253	100

TN-11	chr5	64566043	64570748	CDT1	4705	100
TN-11	chr5	64570035	64640375	PIEZO1	70340	100
TN-11	chr5	64640389	64647215	CTU2	6826	100
TN-11	chr5	64647415	64655999	RNF166	8190	95
TN-11	chr7	30967013	30971096	ENSCAFG00000029072	4083	100
TN-11	chr26	37835661	37913176	PTEN	77086	99
TN-11	chr36	6989488	6997900	TBR1	8233	98
TN-11	chr38	17956961	18109049	LMX1A	150111	99
TN-12	chr27	25779162	25782134	ENSCAFG00000016278	2972	100
TN-12	chr27	25814516	25816336	ENSCAFG00000016289	1820	100
TN-14	chr5	58059031	58146193	MEGF6	86043	99
TN-15	chr23	1864664	1868679	ENSCAFG00000030360	3639	91
TN-16	chr12	16856162	16858711	ENSCAFG00000002106	2549	100
TN-17	chr12	16856162	16858711	ENSCAFG00000002106	2549	100
TN-17	chr16	10347145	10394594	CCM2	45228	95
TN-17	chr27	25712775	25713119	ENSCAFG00000025589	317	92
TN-17	chr27	25749172	25751691	ENSCAFG00000043057	2519	100
TN-17	chr27	25779162	25782134	ENSCAFG00000016278	2972	100
TN-17	chr27	25814516	25816336	ENSCAFG00000016289	1820	100
TN-17	chr32	9993278	9995230	ENSCAFG00000009406	1952	100
TN-18	chr27	25712775	25713119	ENSCAFG00000025589	317	92
TN-18	chr27	25749172	25751691	ENSCAFG00000043057	2519	100
TN-18	chr27	25779162	25782134	ENSCAFG00000016278	2972	100
TN-18	chr27	25814516	25816336	ENSCAFG00000016289	1820	100
TN-19	chr2	28069051	28087760	GATA3	18311	98
TN-19	chr4	22965967	22980292	DNAJB12	14325	100
TN-19	chr4	34589597	34605576	MMRN2	15979	100
TN-19	chr4	34605091	34608842	SNCG	3751	100
TN-19	chr4	34611680	34613754	ADIRF	2074	100
TN-19	chr4	34619615	34621515	ENSCAFG00000016093	1900	100
TN-19	chr4	39588339	39590514	DUSP1	2175	100
TN-19	chr5	4651863	4691069	ST14	38659	99
TN-19	chr5	14900208	14919193	BCL9L	18985	100
TN-19	chr5	14923089	14932762	CXCR5	9673	100
TN-19	chr5	31631838	31657792	CAMTA2	25954	100
TN-19	chr5	31639189	31642914	INCA1	3725	100
TN-19	chr5	31657699	31661420	SPAG7	3721	100
TN-19	chr5	31670194	31671895	PFN1	1701	100
TN-19	chr5	31674284	31676441	RNF167	2157	100
TN-19	chr5	32504695	32507291	MPDU1	2596	100
TN-19	chr6	28465035	28478177	NTAN1	13142	100
TN-19	chr10	62830187	62837172	OTX1	6985	100

TN-19	chr16	6691254	6706207	TRPV6	14953	100
TN-19	chr16	6706313	6718566	EPHB6	12140	99
TN-19	chr16	8346169	8364353	ADCK2	17812	98
TN-19	chr18	52072796	52074336	FAU	1540	100
TN-19	chr22	49948778	49950055	ZIC2	1277	100
TN-19	chr27	1229600	1231359	ENSCAFG00000028496	1759	100
TN-19	chr27	1234689	1236137	HOXC6	1448	100
TN-19	chr27	1252730	1254876	HOXC8	2146	100
TN-19	chr27	2096616	2113619	TNS2	17003	100
TN-19	chr30	39930300	39960566	TMEM266	30266	100
TN-20	chr21	28025759	28026709	OR52A24	950	100
TN-20	chr21	28033762	28034643	OR52AE1	881	100
TN-20	chr27	25712775	25713119	ENSCAFG00000025589	317	92
TN-20	chr27	25749172	25751691	ENSCAFG00000043057	2519	100
TN-20	chr27	25779162	25782134	ENSCAFG00000016278	2972	100
TN-20	chr27	25814516	25816336	ENSCAFG00000016289	1820	100
TN-21	chr5	56503720	56506898	TAS1R3	3178	100
TN-21	chr10	28063624	28122953	MYH9	59329	100
TN-21	chr18	49090689	49104051	MRPL21	13362	100
TN-21	chr19	19855013	19935019	ENSCAFG00000012482	79951	100
TN-22	chr5	30526720	30539483	MYBBP1A	12763	100
TN-22	chr5	30540450	30543773	GGT6	3323	100
TN-22	chr26	37835661	37913176	PTEN	77515	100
TN-23	chr9	5433495	5438372	ENSCAFG00000004703	4877	100
TN-23	chr21	26819309	26820256	OR52P2	947	100
TN-23	chr21	26847230	26848138	OR52K1B	908	100
TN-23	chr29	41768470	41769408	OR6C75C	938	100
TN-24	chr6	17393145	17394707	ENSCAFG00000042608	1562	100
TN-24	chrX	40744408	40746837	NDUFB11	2365	97
TN-24	chrX	40747178	40776742	RBM10	29564	100
TN-24	chrX	40783809	40807069	UBA1	23260	100
TN-24	chrX	40810500	40842700	USP11	32113	100
TN-24	chrX	40844142	40845143	ENSCAFG00000015024	1001	100
TN-24	chrX	40952636	41005133	ZNF157	51997	99
TN-24	chrX	40961826	40962622	ENSCAFG00000015042	720	90
TN-25	chr2	68892492	68901031	MARCKSL1	8539	100
TN-25	chr11	73907111	73925270	ENSCAFG00000041427	17682	97
TN-25	chr16	15111116	15126617	SLC4A2	15501	100
TN-25	chr27	1234689	1236137	HOXC6	1448	100
TN-25	chr27	1252730	1254876	HOXC8	2146	100
TN-26	chr2	28069051	28087760	GATA3	18311	98
TN-26	chr5	32202825	32207000	PHF23	4175	100

TN-26	chr5	32207887	32209908	GABARAP	2021	100
TN-26	chr5	32211343	32216698	CTDNEP1	5355	100
TN-26	chr5	32217464	32223732	ELP5	6268	100
TN-26	chr5	32224040	32226168	CLDN7	2128	100
TN-26	chr5	32238095	32244021	SLC2A4	5386	91
TN-26	chr5	41663773	41686776	SREBF1	23003	100
TN-26	chr5	67001022	67180979	GSE1	164651	91
TN-26	chr26	13645511	13689367	TESC	43856	100
TN-27	chr2	35981105	35981608	ENSCAFG00000041987	503	100
TN-27	chr3	91812875	91849446	PIGG	33775	92
TN-27	chr5	30135759	30235865	ZZEF1	99237	99
TN-27	chr5	30526720	30539483	MYBBP1A	12763	100
TN-27	chr5	30540450	30543773	GGT6	3323	100
TN-27	chr5	56172446	56185932	NOC2L	13486	100
TN-27	chr5	56187922	56192983	KLHL17	5061	100
TN-27	chr5	56194583	56202293	PLEKHN1	7710	100
TN-27	chr5	56202939	56204453	PERM1	1514	100
TN-27	chr5	56225497	56226772	HES4	1275	100
TN-27	chr5	56239701	56272745	AGRN	33044	100
TN-27	chr5	56283491	56287251	ENSCAFG00000028544	3760	100
TN-27	chr10	1352470	1353321	NXPH4	851	100
TN-28	chr3	15707640	15716875	NR2F1	8872	96
TN-28	chr5	4651863	4691069	ST14	39206	100
TN-28	chr7	497394	502952	SYT2	5558	100
TN-28	chr8	70946994	70955867	EXOC3L4	8873	100
TN-29	chr2	81196463	81215689	ATP13A2	19226	100
TN-29	chr2	81220431	81224243	MFAP2	3812	100
TN-29	chr2	84120945	84121388	ENSCAFG00000045358	443	100
TN-29	chr25	51624447	51628668	ENSCAFG00000013188	4221	100
TN-29	chr35	24448828	24449455	ENSCAFG00000010990	627	100
TN-29	chr35	24483501	24486056	ENSCAFG00000047468	2555	100
TN-29	chr35	24499396	24500998	ENSCAFG00000045422	1602	100
TN-29	chr35	24544083	24544475	H2AC11	392	100
TN-30	chr2	80869369	80885928	RCC2	16559	100
TN-30	chr2	81196463	81215689	ATP13A2	19226	100
TN-30	chr2	81220431	81224243	MFAP2	3812	100
TN-30	chr2	81224352	81263550	CROCC	37246	95
TN-30	chr13	2440507	2442675	ENSCAFG00000043864	2168	100
TN-31	chr21	26819309	26820256	OR52P2	947	100
TN-31	chr21	26847230	26848138	OR52K1B	908	100
TN-32	chr19	19855013	19935019	ENSCAFG00000012482	72915	91
TN-32	chr34	12398370	12399773	FOXN3	1273	91

TN-34	chr30	751419	752048	ENSCAFG00000047951	629	100
TN-35	chr18	11332642	11567476	ENSCAFG00000041010	223539	95
TN-35	chr18	11446224	11475118	ENSCAFG00000044110	28894	100
TN-35	chr23	1864664	1868679	ENSCAFG00000030360	3639	91
TN-35	chr29	7185678	7187043	RAB28	1365	100
TN-36	chr26	37835661	37913176	PTEN	75766	98
TN-37	chr27	25712775	25713119	ENSCAFG00000025589	317	92
TN-37	chr27	25749172	25751691	ENSCAFG00000043057	2519	100
TN-37	chr27	25779162	25782134	ENSCAFG00000016278	2972	100
TN-37	chr27	25814516	25816336	ENSCAFG00000016289	1820	100
TN-37	chr29	41768470	41769408	OR6C75C	938	100
TN-38	chr27	25779162	25782134	ENSCAFG00000016278	2972	100
TN-38	chr27	25814516	25816336	ENSCAFG00000016289	1820	100
TN-38	chrX	120910842	120912251	PNMA6A	1409	100
TN-38	chrX	121013351	121015333	ENSCAFG00000019182	1982	100
TN-38	chrX	121031035	121042030	ENSCAFG00000023819	10995	100
TN-41	chr2	35981105	35981608	ENSCAFG00000041987	503	100
TN-41	chr8	2323009	2323657	ENSCAFG00000028548	648	100
TN-41	chr16	1310547	1315763	ENSCAFG00000036837	5216	100
TN-43	chr2	84120945	84121388	ENSCAFG00000045358	443	100
TN-43	chr12	16856162	16858711	ENSCAFG00000002106	2549	100
TN-43	chr16	54054746	54056353	ENSCAFG00000049688	1607	100
TN-43	chr27	25712775	25713119	ENSCAFG00000025589	317	92
TN-43	chr27	25749172	25751691	ENSCAFG00000043057	2519	100
TN-43	chr27	25779162	25782134	ENSCAFG00000016278	2972	100
TN-43	chr27	25814516	25816336	ENSCAFG00000016289	1820	100
TN-44	chr2	81224352	81263550	CROCC	37246	95
TN-44	chr2	84120945	84121388	ENSCAFG00000045358	443	100
TN-45	chr27	25749172	25751691	ENSCAFG00000043057	2519	100
TN-45	chr27	25779162	25782134	ENSCAFG00000016278	2972	100
TN-45	chr27	25814516	25816336	ENSCAFG00000016289	1737	95
TN-46	chr16	54099810	54101447	ENSCAFG00000045217	1637	100
TN-46	chr27	25712775	25713119	ENSCAFG00000025589	317	92
TN-46	chr27	25749172	25751691	ENSCAFG00000043057	2519	100
TN-46	chr27	25779162	25782134	ENSCAFG00000016278	2972	100
TN-46	chr27	25814516	25816336	ENSCAFG00000016289	1820	100
TN-50	chr21	29997525	29998802	ENSCAFG00000006595	1277	100
TN-51	chr3	38149309	38149794	ENSCAFG00000024415	466	96
TN-51	chr23	1864664	1868679	ENSCAFG00000030360	3997	100
TN-51	chr27	25712775	25713119	ENSCAFG00000025589	317	92
TN-51	chr27	25749172	25751691	ENSCAFG00000043057	2519	100
TN-51	chr27	25779162	25782134	ENSCAFG00000016278	2972	100

TN-51	chr27	25814516	25816336	ENSCAFG00000016289	1820	100
TN-51	chr29	41768470	41769408	OR6C75C	938	100
TN-52	chr1	107947573	107957921	ZNF114	10348	100
TN-52	chr1	115088302	115090482	ENSCAFG00000030354	2180	100
TN-52	chrX	41818950	41825811	WDR13	6861	100
TN-54	chr10	17315015	17318512	ZBED4	3497	100
TN-54	chr27	25712775	25713119	ENSCAFG00000025589	317	92
TN-54	chr27	25749172	25751691	ENSCAFG00000043057	2519	100
TN-54	chr27	25779162	25782134	ENSCAFG00000016278	2972	100
TN-54	chr27	25814516	25816336	ENSCAFG00000016289	1820	100
TN-55	chr27	25779162	25782134	ENSCAFG00000016278	2972	100

Supplementary Table S3 b) Summary of genetic deletions in the dataset overlapping >90% of the gene transcript. The canine reference assembly CanFam3.1 was used for the alignment and annotation.
