# Supplementary material for: PIK3CA is recurrently mutated in canine mammary tumors, similarly to in human mammary neoplasia
Source: Sci Rep. 2023 Jan 12;13:632. doi: 10.1038/s41598-023-27664-7 (PMC9837039; doi:10.1038/s41598-023-27664-7)
Supplement: Supplementary file 4 — Supplementary Information 4. [file 41598_2023_27664_MOESM4_ESM.pdf]

## Supplementary Table S4

| Gene | Transcript | Chromosome | Position | Protein change | REF | ALT | Reference | Consequence | SIFT score | PhyloP | AF |
|---|---|---|---|---|---|---|---|---|---|---|---|
| BRCA1 | ENSCAFT00000065506.1 | CHR:9 | 19985052 | p.G282S | G | A | * | MISSENSE | 1.00 | -0.442 | 0.05 |
| BRCA1 | ENSCAFT00000065506.1 | CHR:9 | 19986007 | p.G600D | G | A | * | MISSENSE | 0.09 | -1.674 | 0.02 |
| BRCA1 | ENSCAFT00000065506.1 | CHR:9 | 19987314 | P.C1036R | T | C | * | MISSENSE | 0.29 | -0.143 | 0.03 |
| BRCA1 | ENSCAFT00000065506.1 | CHR:9 | 19987956 | p.T1250A | A | G | * | MISSENSE | 1.00 | -0.128 | 0.05 |
| BRCA1 | ENSCAFT00000065506.1 | CHR:9 | 19988291 | | G | A | Borge et al 2011 , Illumina 170K Canine HD array (BICF2G630829453), * | SYNONYMOUS | | 0.724 | 0.67 |
| BRCA1 | ENSCAFT00000065506.1 | CHR:9 | 19988343 | p.P1379R | G | A | * | MISSENSE | 0.18 | 0.572 | 0.05 |
| BRCA1 | ENSCAFT00000065506.1 | CHR:9 | 20000085 | p.D1521E | C | A | * | MISSENSE | 0 | 1.551 | 0.04 |
| BRCA1 | ENSCAFT00000023190.5 | CHR:9 | 20022197 | p.S1743RX | A | AG | * | FRAMESHIFT | | | 0.04 |
| BRCA1 | ENSCAFT00000023190.5 | CHR:9 | 20022212 | p.W1748R | T | C | * | MISSENSE | | 1.231 | 0.04 |
| BRCA1 | ENSCAFT00000065506.1 | CHR:9 | 20024358 | p.A1912T | G | A | * | MISSENSE | 0.04 | 1.444 | 0.04 |

**Supplementary Table S4 a)** Summary of the germ-line variants found in the *BRCA1* gene in the normal samples in the study. Heat map highlights the SIFT, phyloP scores and allele frequency for each variant. More intense red color denotes lower SIFT score, higher phyloP score and higher allele frequency in the population. Blue color denotes low phyloP scores indicating low levels of constraint. References refer to studies which have previously described the variant in dogs. Star indicates that the variant was found in the current study. The canine reference assembly CanFam3.1 was used for the alignment and annotation.

| Gene | Transcript | Chromosome | Position | Protein change | REF | ALT | Reference | Consequence | SIFT score | PhyloP | AF |
|---|---|---|---|---|---|---|---|---|---|---|---|
| BRCA2 | ENSCAFT00000010309.4 | CHR:25 | 7735412 | p.M3332IK | C | CTTT | Yoshikawa et al 2005, Borge et al 2011, Huskey et al 2020, * | INSERTION | | | 0.82 |
| BRCA2 | ENSCAFT00000010309.4 | CHR:25 | 7748722 | p.N2929D | T | C | * | MISSENSE | 0.01 | 0.037 | 0.01 |
| BRCA2 | ENSCAFT00000010309.4 | CHR:25 | 7753884 | | A | G | * | SYNONYMOUS | | 1.064 | 0.06 |
| BRCA2 | ENSCAFT00000010309.4 | CHR:25 | 7768681 | | G | A | Yoshikawa et al 2008, Borge et al 2011, Huskey et al 2020, * | SYNONYMOUS | | 3.71 | 0.12 |
| BRCA2 | ENSCAFT00000010309.4 | CHR:25 | 7768690 | p.LL2307L | TAAC | T | Borge et al 2011, Huskey et al 2020, * | IN FRAME DELETION | | | 0.08 |
| BRCA2 | ENSCAFT00000010309.4 | CHR:25 | 7770765 | p.C2229F | C | A | * | MISSENSE | 0.35 | 0.707 | 0.04 |
| BRCA2 | ENSCAFT00000010309.4 | CHR:25 | 7770789 | p.P2221L | G | A | * | MISSENSE | 0 | 4.606 | 0.02 |
| BRCA2 | ENSCAFT00000010309.4 | CHR:25 | 7773147 | p.K1435R | T | C | Yoshikawa et al 2008, Huskey et al 2020, Illumina HD SNP TIGRP2P324166, * | MISSENSE | 0.07 | 1.325 | 0.25 |
| BRCA2 | ENSCAFT00000010309.4 | CHR:25 | 7773178 | p.T1425P | T | G | Yoshikawa et al 2008, * | MISSENSE | 0 | 3.664 | 0.05 |
| BRCA2 | ENSCAFT00000010309.4 | CHR:25 | 7775050 | p.K801Q | T | G | Borge et al 2011, Huskey et al 2020 * | MISSENSE | 0.05 | 0.491 | 0.27 |
| BRCA2 | ENSCAFT00000010309.4 | CHR:25 | 7775229 | p.N741S | T | C | Huskey et al 2020, * | MISSENSE | 1 | 1.298 | 0.98 |
| BRCA2 | ENSCAFT00000010309.4 | CHR:25 | 7775268 | p.A728V | G | A | Huskey et al 2020, * | MISSENSE | 0.28 | -0.182 | 0.96 |
| BRCA2 | ENSCAFT00000010309.4 | CHR:25 | 7775297 | | G | T | Huskey et al 2020, * | SYNONYMOUS | | 0.012 | 0.91 |
| BRCA2 | ENSCAFT00000010309.4 | CHR:25 | 7775307 | p.P715Q | G | T | Huskey et al 2020, * | MISSENSE | 1 | -0.151 | 0.91 |
| BRCA2 | ENSCAFT00000010309.4 | CHR:25 | 7777317 | p.F428L | A | G | Yoshikawa et al 2008, Huskey et al 2020 | MISSENSE | 0.29 | 1.434 | 0.01 |
| BRCA2 | ENSCAFT00000010309.4 | CHR:25 | 7777441 | p.C386W | A | C | Yoshikawa et al 2008, * | MISSENSE | 1 | 1.986 | 0.72 |
| BRCA2 | ENSCAFT00000010309.4 | CHR:25 | 7787056 | p.H143R | T | C | Yoshikawa et al 2008, Borge et al 2011, Huskey et al 2020, Illumina HD SNP BICF2P918004, * | MISSENSE | 0.27 | -0.386 | 0.11 |
| BRCA2 | ENSCAFT00000010309.4 | CHR:25 | 7787802 | p.I103T | A | G | * | MISSENSE | 0.22 | -0.278 | 0.05 |

**Supplementary Table S4 b)** Summary of the germ-line variants found in the *BRCA2* gene in the normal samples in the study. Heat map highlights the SIFT, phyloP scores and allele frequency for each variant. More intense red color denotes lower SIFT score, higher phyloP score and higher allele frequency in the population. Blue color denotes low phyloP scores indicating low levels of constraint. References refer to studies which have previously described the variant in dogs. Star indicates that the variant was found in the current study. The canine reference assembly CanFam3.1 was used for the alignment and annotation.
